# The effects of Panax notoginseng for the treatment of diabetic nephropathy in animals: a systematic review and meta-analysis

**DOI:** 10.3389/fnut.2026.1780933

**Published:** 2026-03-17

**Authors:** Jiangteng Liu, Ying Tang, Yuanyuan Lin, Zhichao Ruan, Zhixun Guo, Yexin Chen, Yixin Ma, Weijun Huang, Jinxi Zhao

**Affiliations:** 1 Dongzhimen Hospital, Beijing University of Chinese Medicine, Beijing, China; 2 Key Laboratory of Chinese Internal Medicine of Ministry of Education and Beijing, Beijing, China

**Keywords:** diabetic nephropathy, Panax notoginseng, animal studies, meta-analysis, systematic review

## Abstract

**Background:**

Diabetic nephropathy (DN) is a major microvascular complication of diabetes and constitutes a leading cause of end-stage renal disease (ESRD). Panax notoginseng is a widely utilized traditional Chinese medicinal herb with diverse pharmacological properties, including antioxidant, antithrombotic, and glucolipid metabolism-regulating activities. Preclinical studies have demonstrated the renoprotective effects of Panax notoginseng in DN animal models. However, a comprehensive meta-analysis of these studies is currently lacking, and the dose-time-response relationship remains unexplored.

**Objective:**

This study aimed to systematically evaluate the efficacy of Panax notoginseng in animal models of DN and, for the first time, to explore its dose-time-response relationship. Additionally, we sought to summarize the potential mechanisms underlying its therapeutic effects on DN.

**Methods:**

A systematic search was conducted across seven databases: PubMed, Web of Science, Embase, Chinese Biomedical Database (CBM), CNKI, WanFang, and VIP. The methodological quality of the included studies was assessed using SYRCLE’s risk of bias tool. Statistical analyses were performed using STATA 14.0 software. Primary outcomes included fasting blood glucose (FBG), serum creatinine (SCr), blood urea nitrogen (BUN), 24-h urinary protein (24 h UPro), and kidney index (KI). Secondary outcomes encompassed indicators related to inflammatory response, oxidative stress, glucose and lipid metabolism, and fibrosis. Where substantial heterogeneity was present, sensitivity and subgroup analyses were conducted to explore its sources. Publication bias was assessed via Egger’s test and funnel plots. The dose-time-response relationship of Panax notoginseng for DN was evaluated using a three-dimensional surface plot analysis.

**Results:**

A total of 37 studies were included in the final meta-analysis. The results demonstrated that Panax notoginseng significantly ameliorated FBG, SCr, BUN, 24 h UPro, and KI levels. Furthermore, Panax notoginseng significantly modulated key inflammatory markers, including interleukin-1β (IL-1β), interleukin-6 (IL-6), tumor necrosis factor-*α* (TNF-α), and monocyte chemoattractant protein-1 (MCP-1). It also altered oxidative stress markers, such as superoxide dismutase (SOD) and malondialdehyde (MDA). In addition, Panax notoginseng exerted beneficial effects on lipid metabolism, as evidenced by reduced levels of total cholesterol (TC) and triglycerides (TG). It also attenuated the expression of the pro-fibrotic marker transforming growth factor-β1 (TGF-β1). The three-dimensional dose-time-response analysis revealed that treatment with PNS at a dosage of 20–200 mg/kg/d for 8–12 weeks conferred optimal therapeutic benefits in the DN models.

**Conclusion:**

Panax notoginseng may delay the progression of DN through multiple pathways, including anti-inflammatory, antioxidant, glucolipid metabolism-regulating, and anti-fibrotic mechanisms. However, further studies are still needed to verify its efficacy and mechanisms.

**Systematic review registration:**

https://www.crd.york.ac.uk/PROSPERO/view/CRD420250653856.

## Introduction

Diabetic nephropathy (DN) is the most severe chronic complication of diabetes mellitus (DM) and the leading cause of end-stage renal disease (1). The global prevalence and incidence of DN have been steadily increasing, contributing to a substantial and growing burden on healthcare systems (2). The pathogenesis of DN is multifactorial, involving a complex interplay of hemodynamic abnormalities, chronic inflammation, oxidative stress, metabolic dysregulation, and fibrosis, among other factors (3). Current mainstay pharmacotherapies for DN include angiotensin-converting enzyme inhibitors (ACEIs), angiotensin II receptor blockers (ARBs), sodium-glucose cotransporter-2 inhibitors (SGLT2is), glucagon-like peptide-1 receptor agonists (GLP-1RAs), and the non-steroidal mineralocorticoid receptor antagonist finerenone (3–6). However, the utility of these agents can be limited by renal function thresholds and complex drug–drug interactions. Furthermore, they are associated with potential adverse effects, including urinary tract infections and diabetic ketoacidosis, among others (6, 7). Consequently, there is a compelling need to explore safe and effective therapeutic alternatives derived from natural products.

Derived from the root of *Panax notoginseng (Burk.) F. H. Chen (Araliaceae)*, Panax notoginseng is a prominent traditional Chinese medicinal herb with a documented history of over four centuries (8). It is believed to promote blood circulation, resolve stasis, stop bleeding, alleviate swelling, and relieve pain. Clinically, it is frequently employed in managing diverse conditions, including cardiovascular and cerebrovascular diseases, diabetes mellitus, chronic kidney disease, and hematological disorders. These applications are largely attributed to its primary bioactive constituents, Panax notoginseng saponins (PNS) (8, 9). PNS comprises a complex mixture of triterpenoid saponins, with notoginsenoside R1 (NGR1), ginsenoside Rb1 (GRb1), and ginsenoside Rg1 (GRg1) being the most predominant and well-characterized (9, 10). In the treatment of DN, PNS has been shown to enhance insulin sensitivity, modulate lipid metabolism, and mitigate inflammation and oxidative stress, thereby alleviating the progression of DN and protecting renal function (9, 11–13). Furthermore, individual saponin components, notably NGR1, GRb1, and GRg1, have demonstrated distinct efficacy in lowering FBG and ameliorating diabetic renal injury in preclinical models (9, 14).

Despite these promising findings, the precise molecular mechanisms underlying the renoprotective effects of Panax notoginseng in DN are not fully elucidated. Moreover, a comprehensive systematic review and meta-analysis synthesizing the evidence from preclinical studies on this topic is currently lacking. Therefore, this study aimed to conduct a systematic review and meta-analysis of existing preclinical studies to evaluate the efficacy and potential mechanisms of Panax notoginseng in DN, elucidate its dose-time-response relationship, and provide a robust evidence base to inform the design of future high-quality translational and clinical research.

## Methods

This systematic review and meta-analysis was designed and conducted in accordance with the Preferred Reporting Items for Systematic Reviews and Meta-Analyses (PRISMA) guidelines to ensure methodological rigor, transparency, and completeness (15). The study protocol has been registered in the International Prospective Register of Systematic Reviews (PROSPERO; CRD420250653856).

### Search strategy

Two researchers independently searched for animal studies related to Panax notoginseng in the treatment of DN across seven databases: PubMed, Web of Science, Embase, Chinese Biomedical Database (CBM), CNKI, WanFang, and VIP. The search was conducted up to October 6, 2025. The complete and tailored search strategies for each database are provided in Supplementary Table 1.

### Eligibility criteria

Inclusion criteria: (1) Population: animal models of DN; (2) Intervention: administration of the main active components of Panax notoginseng, namely PNS, NGR1, GRg1, or GRb1; (3) Comparison: control groups receiving no treatment, a placebo, or a non-therapeutic vehicle; and (4) Outcomes: primary outcomes comprised FBG, SCr, BUN, 24 h UPro, and KI. Secondary outcomes encompassed markers of inflammation, oxidative stress, glucolipid metabolism, and fibrosis.

Exclusion criteria: (1) duplicate publications; (2) research types such as reviews, network pharmacology studies, commentaries, or conference abstracts; (3) observational studies, human clinical trials, or *in vitro* experiments; (4) studies where the intervention did not involve Panax notoginseng components, involved combination therapies with other active agents, or lacked an appropriate control group; (5) studies not utilizing animal models of DN; (6) studies for which the full text was inaccessible; or (7) studies with missing, unreported, or incomplete outcome data.

### Data extraction

Data extraction was conducted independently by two reviewers based on the eligibility criteria. Following initial independent screening, the reviewers cross-verified their results. Any discrepancies were resolved through consensus or, if necessary, by arbitration from a third senior researcher. The following data were extracted from the included studies: (1) study characteristics: first author and publication year; (2) animal characteristics: species, strain, sex, body weight, and sample size per group; (3) model induction method; (4) intervention details: specific Panax notoginseng component, batch number documentation or purity required for commercial products, administration route, dosage, and treatment duration; and (5) outcomes including FBG, SCr, BUN, 24 h UPro, KI, IL-1β, IL-6, TNF-*α*, MCP-1, SOD, MDA, TC, TG, and TGF-β1.

The WebPlotDigitizer 4.7 software was used to extract data presented graphically in the studies. When data were reported as the SEM, the SD was calculated using the formula (SD = SEM × $\sqrt{n}$ ) (16). For studies reporting outcomes at multiple time points, only data from the final time point were included in the analysis. This approach was adopted to maintain statistical independence by avoiding unit-of-analysis errors that would arise from including multiple correlated measurements from the same animals. For studies with multiple intervention doses, the means and standard deviations were pooled into a single experimental group using the formulae (Sample size: N_1_ + N_2_, $\text{Mean}:\frac{N_{1}M_{1}+N_{2}M_{2}}{N_{1}+N_{2}},$ $\mathrm{SD}:\sqrt{\frac{\left(N_{1}-1)SD_{1}^{2}+(N_{2}-1)SD_{2}^{2}+\frac{N_{1}N_{2}}{N_{1}+N_{2}}(M_{1}^{2}+M_{2}^{2}-2M_{1}M_{2}\right)}{N_{1}+N_{2}-1}}$ ). For the dose-time-response analysis, data and *p*-values corresponding to the highest dose were selected. Due to the limited number of studies available for other individual components (NGR1, GRg1, and GRb1), this analysis was performed only for PNS.

### Risk of bias assessment

Two reviewers assessed the methodological quality of the studies using SYRCLE’s risk of bias tool (17). This tool evaluates studies across 10 domains pertaining to selection, performance, detection, attrition, reporting, and other potential biases. Disagreements in assessment were resolved through discussion with a third researcher. The results of the quality assessment were visualized using RevMan 5.3 software.

### Statistical analysis

All statistical analyses were performed using STATA 14 software. The standardized mean difference (SMD) and 95% confidence interval (CI) were used to determine the pooled effect size, with *p* < 0.05 considered statistically significant. The SMD was chosen as the effect measure because the included studies used various assays and units to measure the same outcomes, and SMD allows for meaningful combination of such data by expressing treatment effects in standardized units. Heterogeneity was assessed using the I^2^ statistic. If I^2^ > 50% or *p* < 0.1, significant heterogeneity was indicated. In such cases, a random-effects model was employed; otherwise, a fixed-effects model was used. To explore potential sources of heterogeneity, sensitivity and subgroup analyses were conducted. Subgroup analyses were stratified by animal species (mouse or rats), type of DN model (type 1 or type 2), specific Panax notoginseng component (PNS, NGR1, GRg1, or GRb1), and intervention duration (< 8 weeks, 8 weeks ≤ duration < 12 weeks, or ≥ 12 weeks). Funnel plots and Egger’s test were used to assess publication bias, with trim-and-fill analysis performed if necessary. To visually explore the distribution of treatment effects across different doses and intervention durations, we constructed a three-dimensional scatter plot using Origin 2024 software. This visualization displays the *p*-values of primary outcomes from studies at various doses and time points. The purpose of this visualization was to present the distribution of raw data across the dose-time continuum, maintaining transparency regarding underlying data heterogeneity and avoiding any mathematical assumptions about the dose-time-response relationship. Each study contributes equally to the visualization, reflecting the distribution of published evidence. No interpolation, smoothing, surface fitting, or weighting was applied to the data.

## Results

### Study selection

**Figure 1:**
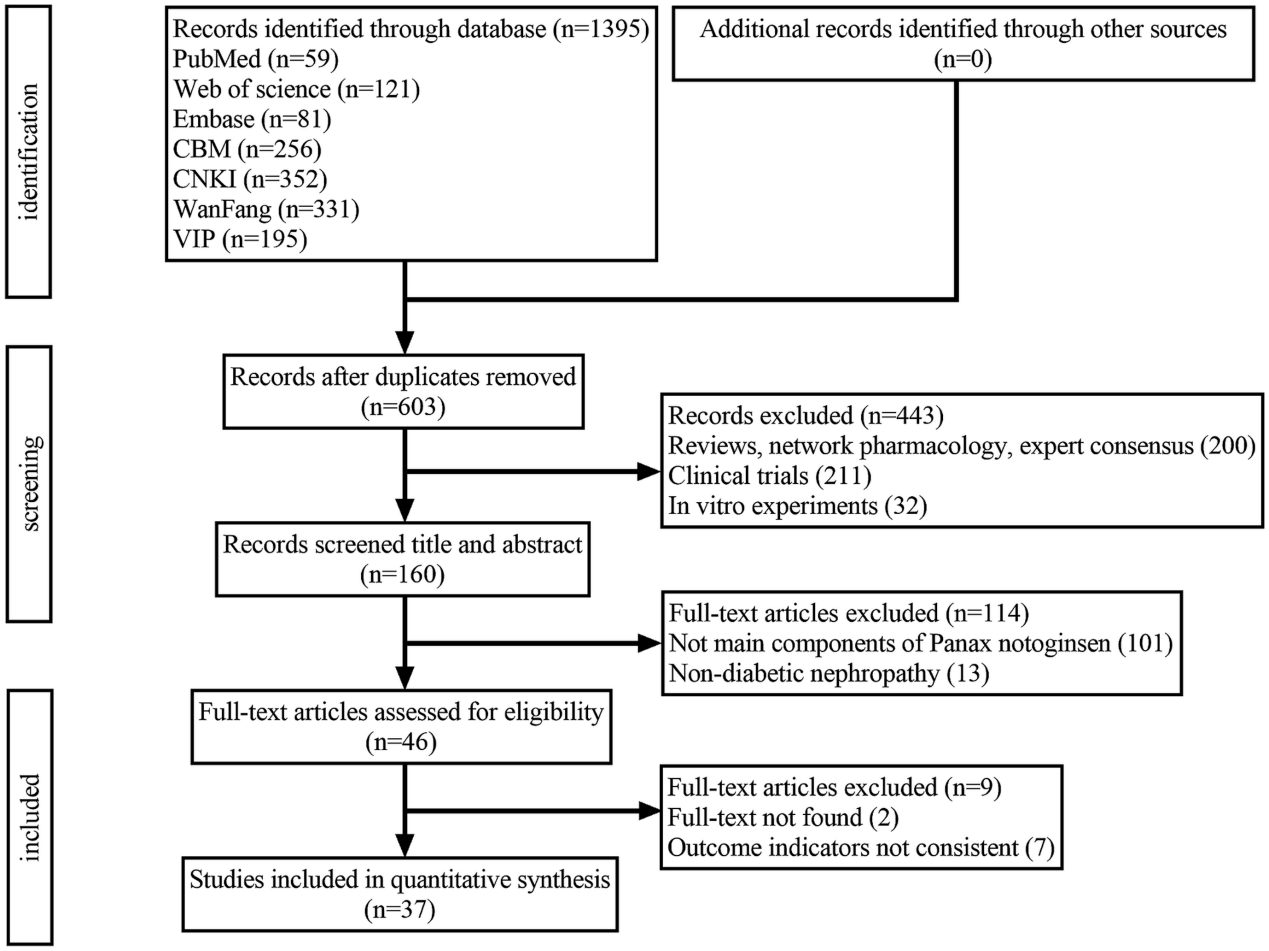
Flow chart of study selection.

A total of 1,395 records were identified across the seven databases. After the removal of 792 duplicate records, 603 unique records remained. Following further screening, 566 studies were excluded, resulting in the final inclusion of 37 studies for the meta-analysis (Figure 1).

### Characteristics of the studies

The 37 included studies involved a total of 910 animals, distributed as 557 in the experimental groups and 353 in the control groups. Regarding animal species and strains, Sprague–Dawley (SD) rats were used in 23 studies, Wistar rats in five studies, C57BL/6 N mice in four studies, and db/db mice in five studies. All studies exclusively utilized male animals. Baseline body weight was reported in 34 studies. The methods for inducing DN varied: five studies employed spontaneous models; 22 studies used streptozotocin (STZ) injection alone; eight studies utilized STZ injection combined with a high-fat diet or a high-fat, high-sugar diet; one study used STZ combined with unilateral subtotal nephrectomy; and 1 study used alloxan. In terms of the modeled DN type, 13 studies focused on type 2 DN, while 24 studies focused on type 1 DN. The Panax notoginseng components used in the studies were commercial products. Manufacturers were documented in 35 studies, with batch numbers or purity specified in 22. The specific Panax notoginseng components investigated were PNS in 22 studies, NGR1 in five studies, GRg1 in nine studies, and GRb1 in only one study. The intervention was administered exclusively via oral gavage in all studies. The intervention duration ranged from four to 20 weeks. For primary outcomes, FBG was reported in 29 studies, SCr in 28, BUN in 25, 24 h UPro in 14, and KI in 16 studies. Of the studies, 30 reported data on various secondary outcome markers related to inflammation, oxidative stress, glucolipid metabolism, and fibrosis (Table 1). The batch numbers or purity on the Panax notoginseng is shown in Supplementary Table 2.

**Table 1:** Study characteristics.

Author, year	Specific component	Species (sex, NE/NC)	Weight	Methods for inducing DN	DN type	Dose	Duration	Outcomes
Chang (40)	PNS	SD rats(male, 10/10)	180–200 g	STZ	I	35 (mg/kg/d)	8 weeks	FBG, SOD, MDA
Chen et al. (32)	PNS	db/db mice(male, 8/8)	38.09 ± 2.11 g	Spont	II	100 (mg/kg/d)	8 weeks	FBG, SCr, BUN, 24 h UPro, KI, TC, TG
Cheng et al. (63)	PNS	Wistar rats(male, 20/10)	180 ± 10 g	STZ + diet	II	100/200 (mg/kg/d)	12 weeks	FBG, SCr, TC, TG
Dong (29)	PNS	C57BL/6 N mice(male, 24/8)	18–22 g	STZ	I	50/100/200 (mg/kg/d)	8 weeks	FBG, SCr, BUN, SOD, MDA
Du et al. (57)	PNS	SD rats(male, 20/10)	180–200 g	Alloxan	I	100/200 (mg/kg/d)	3 months	FBG, SCr, BUN, TC, TG, TGF-β1
Du et al. (51)	GRg1	Wistar rats(male, 8/8)	180–220 g	STZ	I	50 (mg/kg/d)	8 weeks	SCr, BUN, MDA, TGF-β1
Fu et al. (59)	PNS	SD rats(male, 8/8)	150–200 g	STZ	I	100 (mg/kg/d)	8 weeks	FBG, 24 h UPro, KI
Gui et al. (48)	NGR1	SD rats(male, 18/9)	180–200 g	STZ	I	5/10 (mg/kg/d)	12 weeks	FBG, SCr, BUN, 24 h UPro, SOD, MDA
Han et al. (60)	GRg1	C57BL/6 N mice(male, 30/10)	30–40 g	STZ + diet	II	1/5/10 (mg/kg/d)	8 weeks	SCr, BUN, TC, TG, TGF-β1
Hou et al. (24)	PNS	SD rats(male, 10/10)	180 ± 25 g	STZ	I	100 (mg/kg/d)	8 weeks	FBG, SCr, 24 h UPro, TNF-α
Huang et al. (27)	NGR1	SD rats(male, 30/10)	200–220 g	STZ	I	5/10/20 (mg/kg/d)	16 weeks	FBG, SCr, BUN, 24 h UPro, KI, IL-1β, IL-6, TNF-α, MCP-1
Li (45)	PNS	SD rats(male, 12/6)	200–250 g	STZ	I	50/100 (mg/kg/d)	4 weeks	KI, SOD, MDA
Li et al. (42)	PNS	SD rats(male, 11/10)	210–220 g	STZ	I	100 (mg/kg/d)	10 weeks	SCr, BUN, SOD, MDA, TC, TG
Li and Hu (28)	GRg1	SD rats(male, 10/10)	180–200 g	STZ + diet	II	50 (mg/kg/d)	12 weeks	FBG, SCr, BUN, IL-1β, IL-6, TNF-α, SOD, MDA
Li et al. (39)	PNS	SD rats(male, 24/12)	200–220 g	STZ	I	31/62 (mg/kg/d)	8 weeks	FBG, SCr, BUN, KI, SOD, MDA
Li et al. (22)	GRg1	SD rats(male, 14/13)	180–220 g	STZ	I	30 (mg/kg/d)	8 weeks	FBG, SCr, BUN, 24 h UPro, TNF-α, MCP-1, TC, TG
Li (21)	NGR1	SD rats(male, 8/8)	200–220 g	STZ + diet	II	10 (mg/kg/d)	4 weeks	FBG, SCr, BUN, KI, IL-1β, IL-6, TNF-α, MCP-1
Mi et al. (46)	PNS	C57BL/6 N mice(male, 27/9)	20–25 g	STZ + diet	II	50/100/200 (mg/kg/d)	8 weeks	FBG, SCr, BUN, 24 h UPro, SOD, MDA
Peng (55)	PNS	SD rats(male, 9/7)	180–220 g	STZ	I	100 (mg/kg/d)	8 weeks	FBG, SCr, BUN, 24 h UPro, KI, TGF-β1
Qin (56)	PNS	Wistar rats(male, 20/10)	180–200 g	STZ	I	100/200 (mg/kg/d)	6 weeks	FBG, 24 h UPro, KI, TGF-β1
Ruan et al. (71)	GRg1	SD rats(male, 30/10)	180–200 g	STZ + diet	II	25/50/100 (mg/kg/d)	4 weeks	SCr, BUN, KI
Shan (37)	PNS	Wistar rats(male, 15/5)	NR	STZ	I	20/50/100 (mg/kg/d)	10 weeks	FBG, SCr, BUN, KI, SOD, MDA, TC, TG
Shao et al. (72)	GRb1	Wistar rats(male, 10/10)	180–200 g	STZ	I	40 (mg/kg/d)	12 weeks	FBG, SCr, KI, SOD, MDA, TC, TG
Shi and Tu (58)	PNS	SD rats(male, 20/10)	180 ± 10 g	STZ	I	100/200 (mg/kg/d)	4 weeks	FBG, KI
Sun (44)	PNS	SD rats(male, 11/11)	220–230 g	STZ + nephrectomy	I	17.5 (mg/kg/d)	12 weeks	SCr, BUN, 24 h UPro, KI, SOD, MDA, TG, TGF-β1
Wang (41)	PNS	SD rats(male, 10/10)	150–250 g	STZ	I	100 (mg/kg/d)	4 weeks	KI, MDA, TGF-β1
Wang et al. (62)	PNS	db/db mice(male, 20/10)	NR	Spont	II	10/30 (mg/kg/d)	8 weeks	FBG, BUN
Xu et al. (43)	PNS	SD rats(male, 6/6)	150–200 g	STZ	I	100 (mg/kg/d)	8 weeks	SOD, MDA
Xu et al. (38)	PNS	db/db mice(male, 24/12)	30 ± 2	Spont	II	15/20 (mg/kg/d)	8 weeks	FBG, BUN, KI, MDA, TGF-β1
Xue et al. (54)	PNS	SD rats(male, 8/8)	180–200 g	STZ	I	5 (mg/kg/d)	12 weeks	FBG, SCr, BUN, KI
Yang et al. (25)	GRg1	SD rats(male, 20/20)	200 ± 20 g	STZ	I	21 (mg/kg/d)	12 weeks	SCr, BUN, IL-1β, IL-6, TNF-α, SOD, MDA, TGF-β
Zhang et al. (26)	GRg1	SD rats(male, 9/8)	200 ± 20 g	STZ	I	50 (mg/kg/d)	8 weeks	FBG, SCr, 24 h UPro, TNF-α, MCP-1
Zhang et al. (49)	NGR1	db/db mice(male, 8/8)	NR	Spont	II	30 (mg/kg/d)	20 weeks	FBG, SCr, BUN, 24 h UPro, TC, TG, TGF-β
Zhang et al. (50)	NGR1	db/db mice(male, 12/6)	40 ± 2 g	Spont	II	30/60 (mg/kg/d)	8 weeks	FBG, SCr, BUN
Zhang (73)	GRg1	SD rats(male, 8/8)	200–220 g	STZ + diet	II	10 (mg/kg/d)	8 weeks	FBG, SCr, KI, SOD, MDA, TC, TG
Zhang et al. (74)	GRg1	C57BL/6 N mice(male, 12/12)	20–25 g	STZ + diet	II	10 (mg/kg/d)	8 weeks	FBG, SCr, BUN
Zheng et al. (23)	PNS	SD rats(male, 13/13)	180 ± 25 g	STZ	I	100 (mg/kg/d)	8 weeks	FBG, SCr, 24 h UPro, TNF-α, SOD, MDA

### Quality assessment

**Figure 2:**
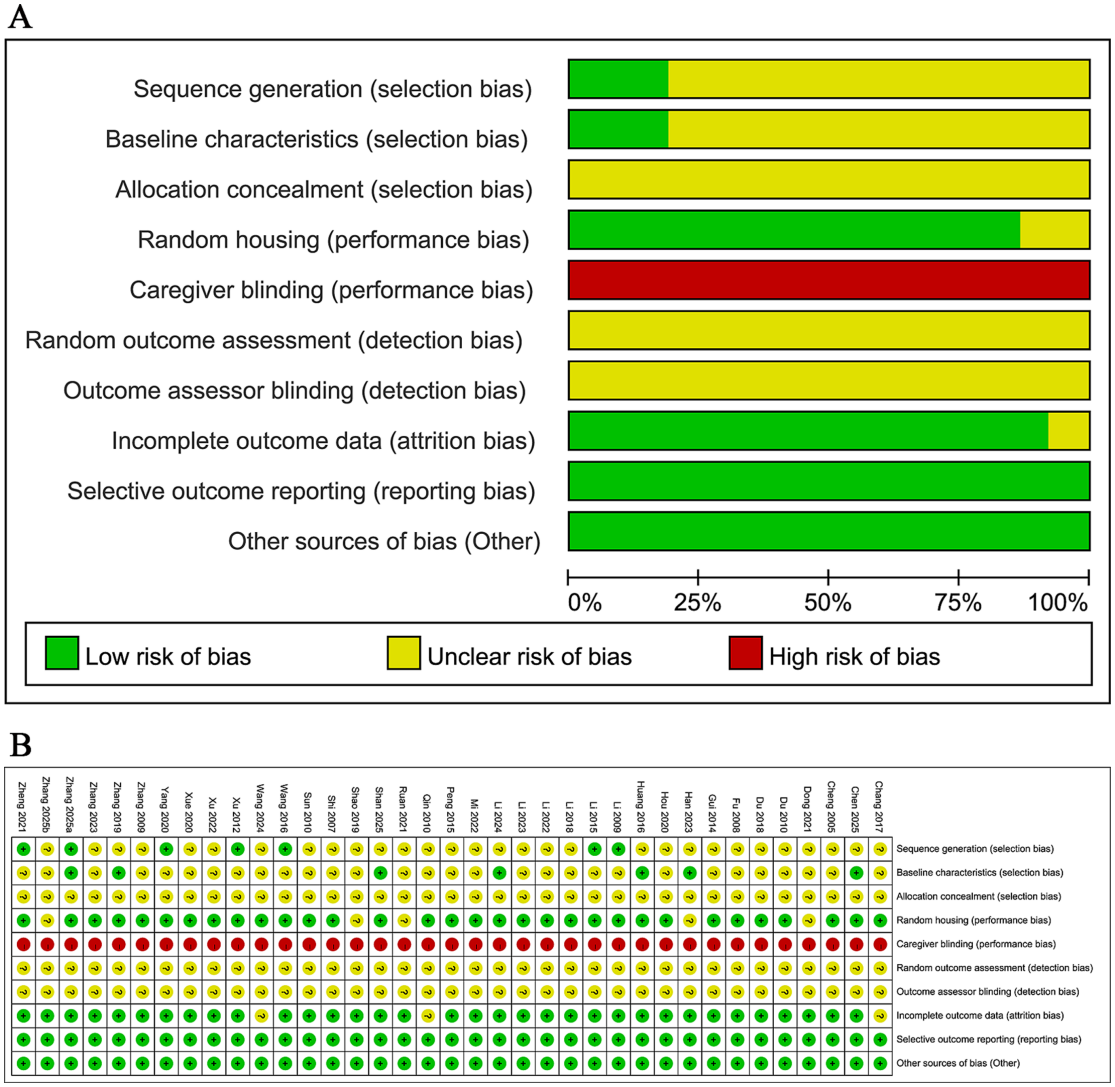
Deviation chart **(A)** and bias summary **(B)** incorporating study risks.

The methodological quality of the studies was assessed using the SYRCLE risk of bias tool and visualized using RevMan 5.3 software (Figure 2A). Overall, the methodological quality of the included studies was moderate to low, with several key domains frequently rated as unclear or high risk. In 37 studies, “Selective outcome reporting” and “Other sources of bias” were judged as low risk, “Blinding of caregivers” was judged as high risk, while “Allocation concealment,” “Random outcome assessment”, and “Blinding of outcome assessors” were of unclear risk. 32 studies reported “Random housing”, seven studies reported “Sequence generation”, seven studies reported “Baseline characteristics”, and three studies had “Incomplete outcome data” (Figure 2B).

### Effectiveness

#### Primary outcomes

##### FBG

**Figure 3:**
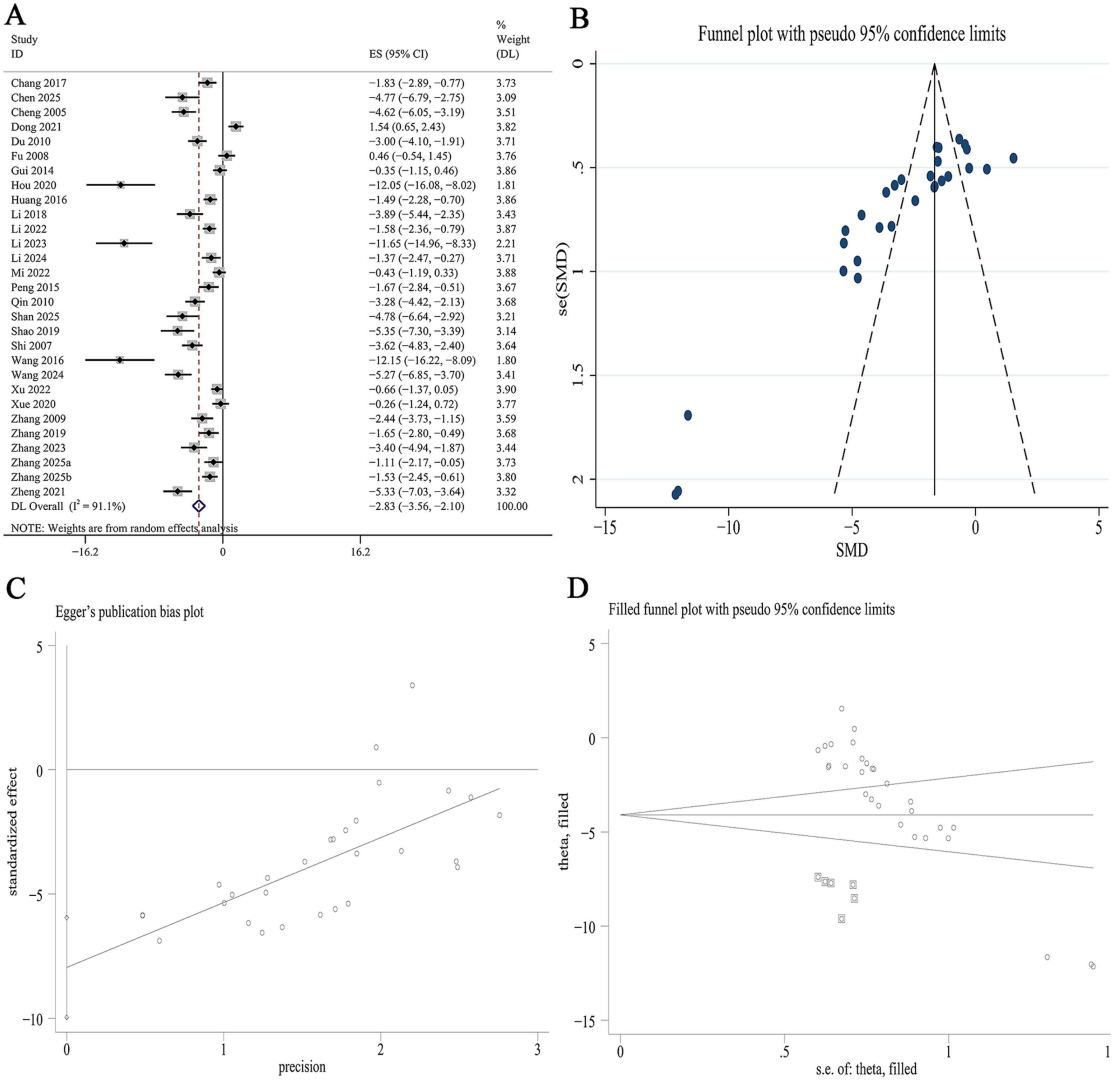
Effect of Panax notoginseng on FBG. **(A)** Forest plot. **(B)** Funnel plot. **(C)** Egger’s publication bias plot. **(D)** Filled funnel plot.

Data on FBG were available from 29 studies. Meta-analysis revealed that Panax notoginseng significantly reduced FBG levels compared to the control [SMD: −2.83, 95% CI (−3.56, −2.10), *p* = 0.000; I^2^ = 91.1%, *p* = 0.000; Figure 3A]. Subgroup analysis indicated that the reduction in FBG was more pronounced in type 1 DN than in type 2 DN (SMD −3.16 vs. −2.46, *p* = 0.028). Stratified by the specific active component, the greatest reduction in FBG was observed with GRb1, followed by GRg1, PNS, and NGR1 (SMD −5.35 vs. −3.53 vs. −2.98 vs. −1.51, *p* = 0.046). Subgroup analysis based on intervention duration demonstrated a significant gradient, with the strongest effect observed in the short-term duration group, followed by the medium-term and long-term groups (SMD −4.18 vs. −2.79 vs. −2.44, *p* = 0.028). No statistically significant difference in FBG reduction was observed between mice and rats (Supplementary Table 2). A funnel plot (Figure 3B) and Egger’s test indicated statistically significant publication bias for FBG (*p* = 0.000; Figure 3C). Trim-and-fill analysis estimated that imputing six theoretically missing studies would yield a symmetric funnel plot (Figure 3D; Supplementary Table 3).

##### SCr

**Figure 4:**
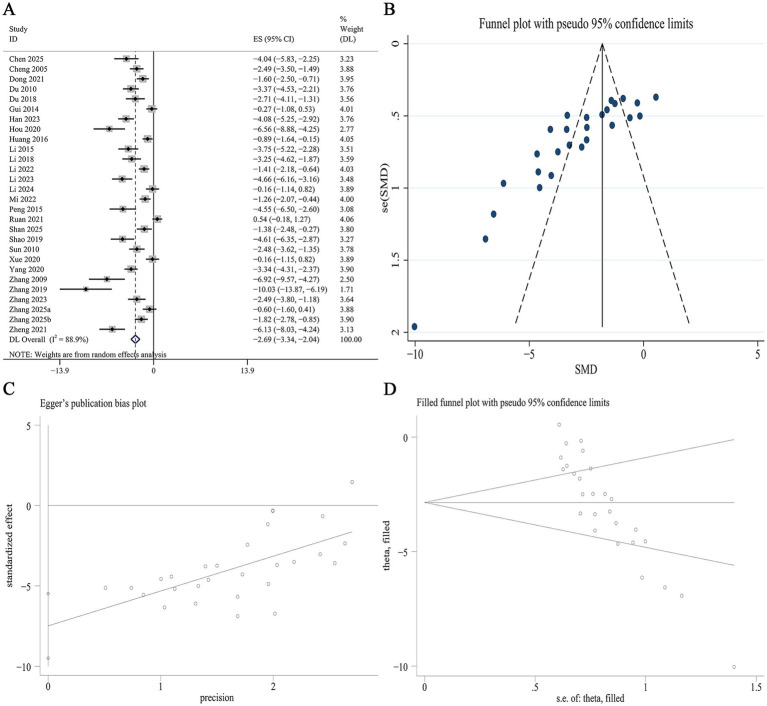
Effect of Panax notoginseng on SCr. **(A)** Forest plot. **(B)** Funnel plot. **(C)** Egger’s publication bias plot. **(D)** Filled funnel plot.

SCr data were reported in 28 studies. Meta-analysis demonstrated a significant reduction in SCr levels with Panax notoginseng compared to control [SMD: −2.69, 95% CI (−3.34, −2.04), *p* = 0.000; I^2^ = 88.94%, *p* = 0.000; Figure 4A]. Subgroup analysis by DN model type revealed a more pronounced reduction in SCr in type 1 DN compared to type 2 DN (SMD −2.98 vs. −2.26, *p* = 0.006). When stratified by active component, GRb1 exhibited the strongest effect, followed by GRg1, PNS, and NGR1 (SMD −4.61 vs. −2.83 vs. −2.77 vs. −1.71, *p* = 0.012). No statistically significant differences in SCr reduction were detected across subgroups of animal species or intervention duration. Notably, heterogeneity was substantially lower in the short-term intervention subgroup (I^2^ = 22.0%, *p* = 0.257; Supplementary Table 2). A funnel plot (Figure 4B) and Egger’s test suggested significant effects, indicating publication bias for SCr outcomes (*p* = 0.000; Figure 4C). Trim-and-fill analysis was subsequently applied to adjust for this asymmetry. The adjusted pooled effect size remained virtually unchanged following imputation, underscoring the robustness of the finding for SCr (Figure 4D).

##### BUN

**Figure 5:**
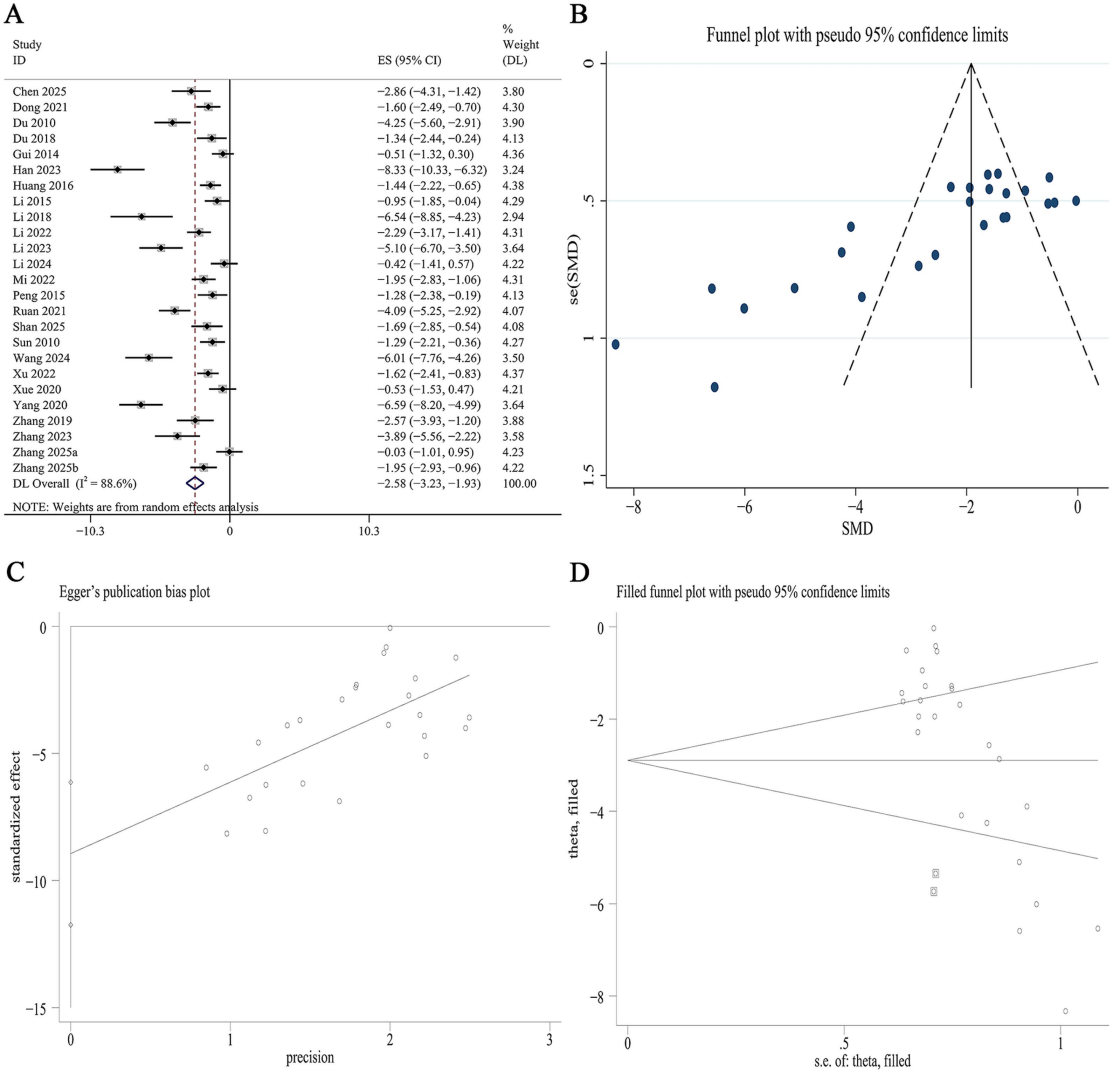
Effect of Panax notoginseng on BUN. **(A)** Forest plot. **(B)** Funnel plot. **(C)** Egger’s publication bias plot. **(D)** Filled funnel plot.

Data on BUN were available from 25 studies. Meta-analysis demonstrated that Panax notoginseng significantly lowered BUN levels compared to control [SMD: −2.58, 95% CI (−3.23, −1.93), *p* = 0.000; I^2^ = 88.6%, *p* = 0.000; Figure 5A]. Subgroup analysis by animal species revealed a more pronounced reduction in BUN in rat models than in mouse models (SMD −3.22 vs. −2.24, *p* = 0.011). No statistically significant differences in BUN reduction were observed across subgroups defined by DN model type, specific active component, or intervention duration (Supplementary Table 2). A funnel plot (Figure 5B) and Egger’s test indicated statistically significant effects, suggesting publication bias for BUN outcomes (*p* = 0.000; Figure 5C). Trim-and-fill analysis estimated that imputing two theoretically missing studies would yield a symmetric funnel plot (Figure 5D; Supplementary Table 3).

##### 24 h UPro

**Figure 6:**
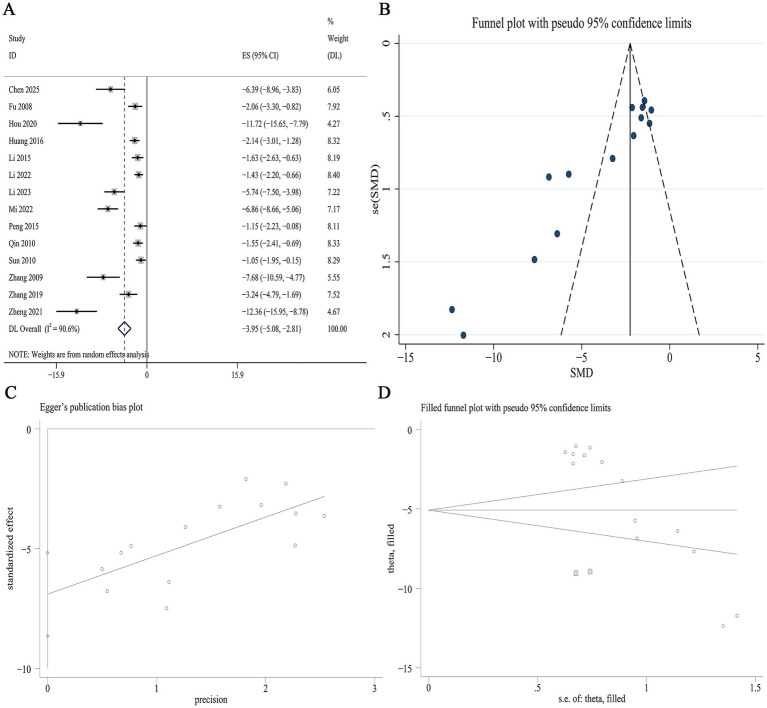
Effect of Panax notoginseng on 24 h UPro. **(A)** Forest plot. **(B)** Funnel plot. **(C)** Egger’s publication bias plot. **(D)** Filled funnel plot.

14 studies reported data on 24 h UPro. Meta-analysis revealed that Panax notoginseng significantly reduced 24 h UPro levels compared to control [SMD: −3.95, 95% CI (−5.08, −2.81), *p* = 0.001; I^2^ = 90.6%, *p* = 0.000; Figure 6A]. Subgroup analyses stratified by different animal species, DN model type, active component, and intervention durations consistently demonstrated beneficial effects, although no statistically significant differences were observed between the subgroups. Notably, heterogeneity was substantially lower in the NGR1 (I^2^ = 32.1%, *p* = 0.225) and GRg1 (I^2^ = 20.2%, *p* = 0.263) subgroups (Supplementary Table 2). A funnel plot (Figure 6B) and Egger’s test indicated statistically significant effects, suggesting the presence of publication bias for 24 h UPro (*p* = 0.000; Figure 6C). Trim-and-fill analysis estimated that imputing two theoretically missing studies would yield a symmetric funnel plot (Figure 6D; Supplementary Table 3).

##### KI

**Figure 7:**
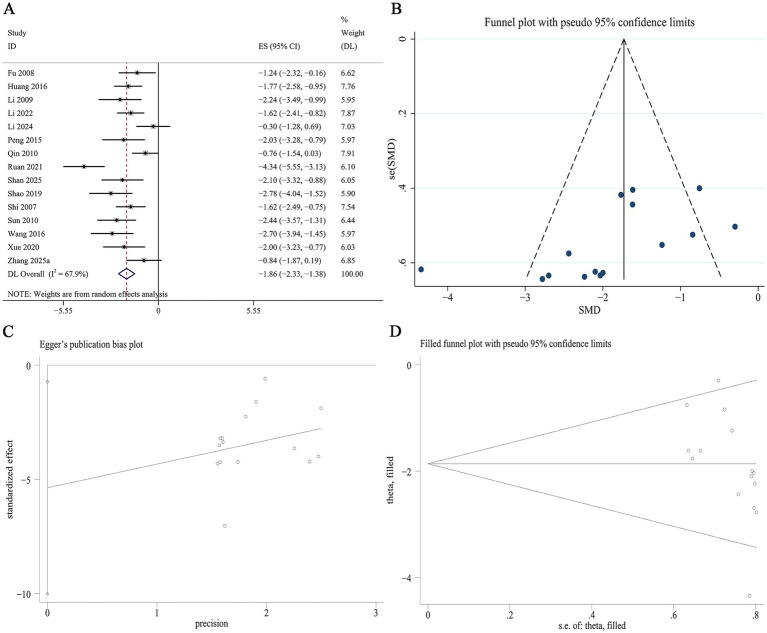
Effect of Panax notoginseng on KI. **(A)** Forest plot. **(B)** Funnel plot. **(C)** Egger’s publication bias plot. **(D)** Filled funnel plot.

16 studies reported data on the KI. Meta-analysis indicated a significant reduction in KI with Panax notoginseng compared to control [SMD: −1.86, 95% CI (−2.33, −1.38), *p* = 0.000; I^2^ = 67.9%, *p* = 0.000; Figure 7A]. Subgroup analysis by DN model type revealed a statistically significantly greater reduction in KI in type 1 DN than in type 2 DN (SMD −1.82 vs. −1.80, *p* = 0.026). When stratified by active component, GRb1 showed the strongest effect, followed by GRg1, PNS, and NGR1 (SMD −2.78 vs. −2.58 vs. −1.76 vs. −1.06, *p* = 0.012). A significant effect of intervention duration was observed, with the long-duration group showing the greatest reduction in KI, followed by the medium-duration and short-duration groups (SMD −2.14 vs. −1.94 vs. −1.52, *p* = 0.027). No statistically significant difference in KI reduction was detected between mice and rats. Heterogeneity was substantially lower in several subgroups, including the type 1 DN model (I^2^ = 25.0%, *p* = 0.198), PNS (I^2^ = 25.5%, *p* = 0.209), medium-term intervention (I^2^ = 0.0%, *p* = 0.478), and long-term intervention (I^2^ = 0.0%, *p* = 0.551) subgroups (Supplementary Table 2). A funnel plot (Figure 7B) and Egger’s test suggested statistically significant effects, indicative of publication bias for KI (*p* = 0.027, Figure 7C). Trim-and-fill analysis was performed to adjust for this potential bias. The adjusted pooled effect size remained virtually unchanged following imputation, confirming the robustness of the finding for KI (Figure 7D).

#### Secondary outcomes

##### Inflammatory biomarkers

Four studies reported data on IL-1β and IL-6, eight studies on TNF-*α*, and three studies on MCP-1. Meta-analysis showed that Panax notoginseng was associated with reductions in IL-1β [SMD: −3.98, 95% CI (−6.64, −1.32), *p* = 0.081; I^2^ = 93.9%, *p* = 0.000], IL-6 [SMD: −2.83, 95% CI (−4.56, −1.10), *p* = 0.054; I^2^ = 89.5%, *p* = 0.000], TNF-α [SMD: −4.55, 95% CI (−6.15, −2.95), *p* = 0.009; I^2^ = 89.4%, *p* = 0.000], and MCP-1 levels [SMD: −6.25, 95% CI (−11.59, −0.91), *p* = 0.155; I^2^ = 94.7%, *p* = 0.000] compared to control. Forest plots are provided in Supplementary Figure 1.

##### Oxidative stress biomarkers

###### Serum SOD

**Figure 8:**
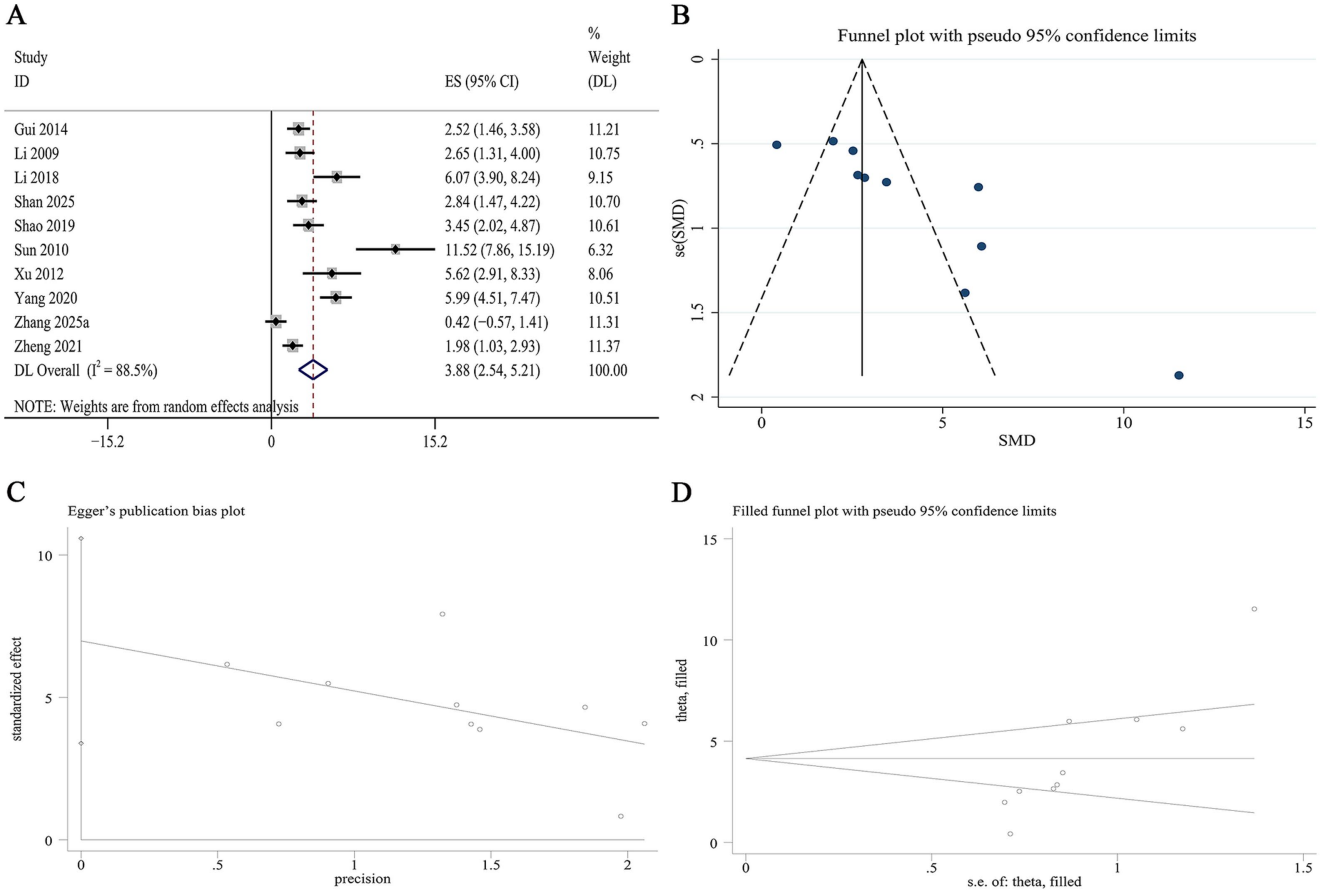
Effect of Panax notoginseng on serum SOD. **(A)** Forest plot. **(B)** Funnel plot. **(C)** Egger’s publication bias plot. **(D)** Filled funnel plot.

10 studies reported data on serum SOD levels. Meta-analysis demonstrated that Panax notoginseng significantly increased serum SOD compared to the control group [SMD: 3.88, 95% CI (2.54, 5.21), *p* = 0.002; I^2^ = 88.5%, *p* = 0.000; Figure 8A]. Subgroup analyses stratified by animal species, DN model type, active component, and intervention duration revealed no statistically significant differences in the magnitude of SOD elevation (Supplementary Table 4). A funnel plot (Figure 8B) and Egger’s test indicated statistically significant effects, suggesting the presence of publication bias for serum SOD (*p* = 0.002; Figure 8C). Trim-and-fill analysis was subsequently performed to adjust for this potential bias. Following imputation, the adjusted pooled effect size remained virtually unchanged, affirming the robustness of the finding regarding SOD (Figure 8D).

###### Renal SOD

**Figure 9:**
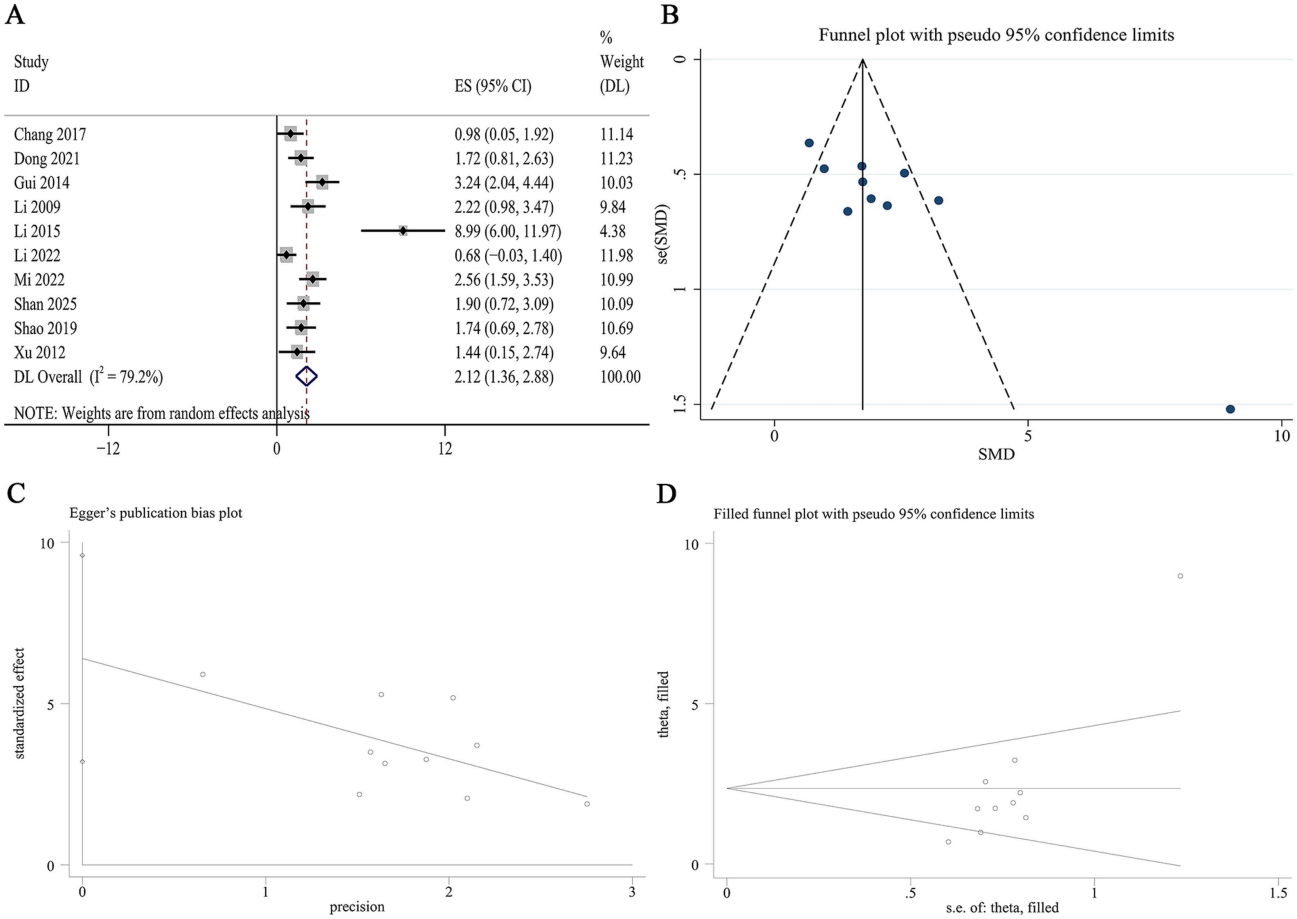
Effect of Panax notoginseng on renal SOD. **(A)** Forest plot. **(B)** Funnel plot. **(C)** Egger’s publication bias plot. **(D)** Filled funnel plot.

10 studies reported renal SOD levels. Meta-analysis indicated that Panax notoginseng significantly increased renal SOD compared to control [SMD: 2.12, 95% CI (1.36, 2.88), *p* = 0.005; I^2^ = 79.2%, *p* = 0.000; Figure 9A]. Subgroup analyses stratified by animal species, DN model type, active component, and intervention duration revealed no statistically significant differences. Notably, heterogeneity was substantially lower in the mouse subgroup (I^2^ = 34.8%, *p* = 0.215; Supplementary Table 4). A funnel plot (Figure 9B) and Egger’s test indicated statistically significant effects, suggesting publication bias for renal SOD outcomes (*p* = 0.002; Figure 9C). Trim-and-fill analysis was subsequently conducted to adjust for this asymmetry. The adjusted pooled effect size remained virtually unchanged following imputation, underscoring the robustness of the result for renal SOD (Figure 9D).

###### Serum MDA

**Figure 10:**
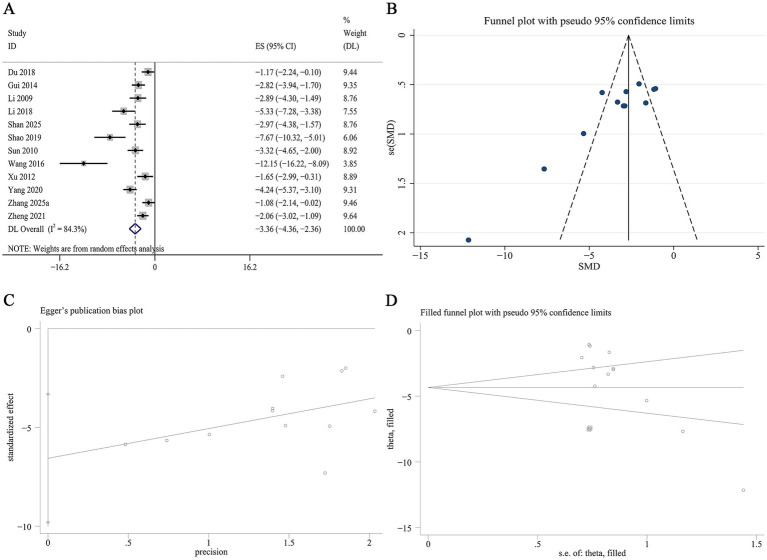
Effect of Panax notoginseng on serum MDA. **(A)** Forest plot. **(B)** Funnel plot. **(C)** Egger’s publication bias plot. **(D)** Filled funnel plot.

12 studies reported data on serum MDA levels. Meta-analysis revealed that Panax notoginseng significantly reduced serum MDA levels compared to control [SMD: −3.36, 95% CI (−4.36, −2.36), *p* = 0.001; I^2^ = 84.3%, *p* = 0.000; Figure 10A]. Subgroup analyses revealed no statistically significant differences in the reduction of serum MDA across subgroups defined by animal species, DN model type, active component, or intervention duration. Notably, heterogeneity was substantially lower in the medium-term intervention subgroup (I^2^ = 36.9%, *p* = 0.191; Supplementary Table 4). A funnel plot (Figure 10B) and Egger’s test indicated statistically significant effects, suggesting publication bias for serum MDA outcomes (*p* = 0.001; Figure 10C). Trim-and-fill analysis estimated that imputing two theoretically missing studies would yield a symmetric funnel plot (Figure 10D; Supplementary Table 3).

###### Renal MDA

**Figure 11:**
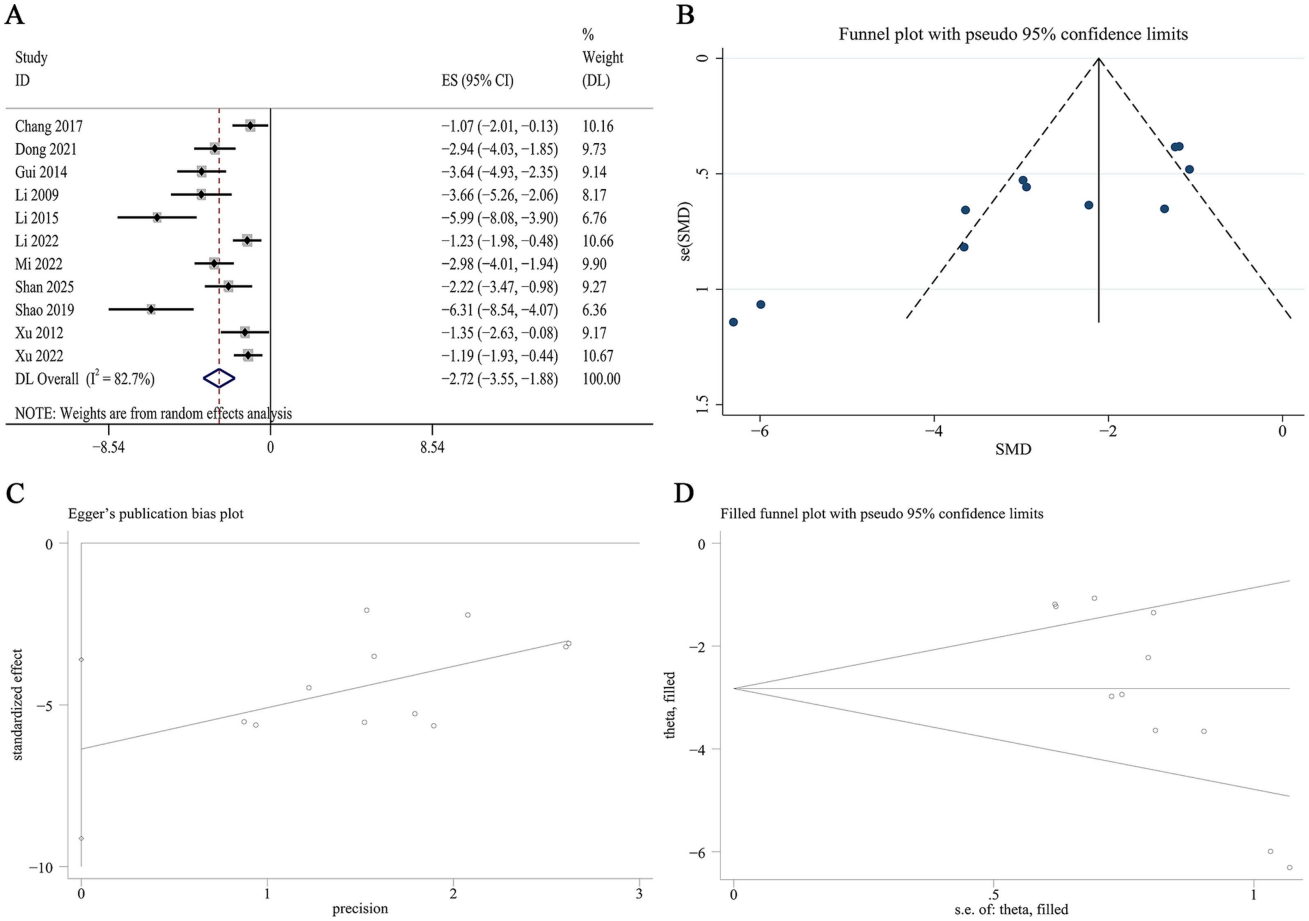
Effect of Panax notoginseng on renal MDA. **(A)** Forest plot. **(B)** Funnel plot. **(C)** Egger’s publication bias plot. **(D)** Filled funnel plot.

11 studies reported data on renal MDA levels. Meta-analysis demonstrated a significant reduction in renal MDA levels with Panax notoginseng compared to control [SMD: −2.72, 95% CI (−3.55, −1.88), *p* = 0.000; I^2^ = 82.7%, *p* = 0.000; Figure 11A]. Subgroup analyses stratified by animal species, DN model type, active component, and intervention duration revealed no statistically significant differences (Supplementary Table 4). A funnel plot (Figure 11B) and Egger’s test indicated statistically significant effects, suggesting publication bias for renal MDA outcomes (*p* = 0.001; Figure 11C). Trim-and-fill analysis was subsequently conducted to adjust for this potential bias. Following imputation, the adjusted pooled effect size remained virtually unchanged, affirming the robustness of the finding for renal MDA (Figure 11D).

##### Glucose and lipid metabolism markers

###### TC

**Figure 12:**
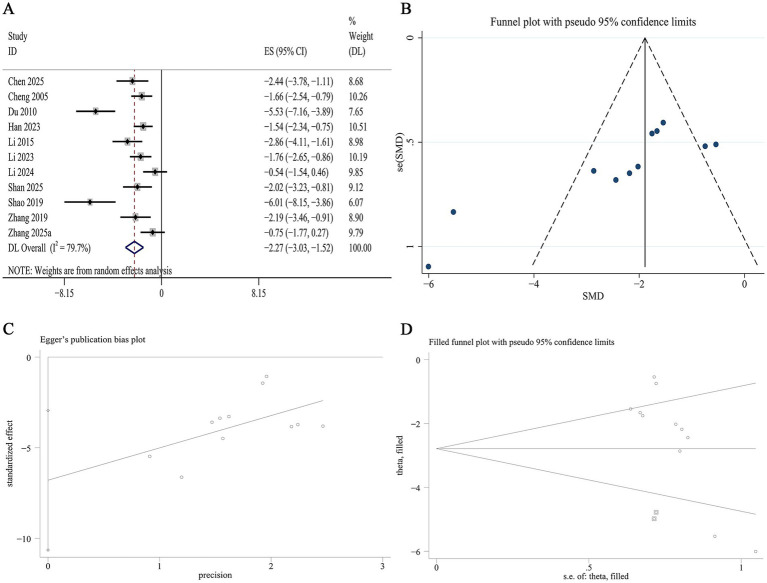
Effect of Panax notoginseng on TC. **(A)** Forest plot. **(B)** Funnel plot. **(C)** Egger’s publication bias plot. **(D)** Filled funnel plot.

11 studies reported data on total TC levels. Meta-analysis revealed that Panax notoginseng significantly reduced TC levels compared to control [SMD: −2.27, 95% CI (−3.03, −1.52), *p* = 0.001; I^2^ = 79.7%, *p* = 0.000; Figure 12A]. Subgroup analysis by DN model type demonstrated a significantly greater reduction in TC in type 1 DN than in type 2 DN (SMD −3.16 vs. -2.46, *p* = 0.004). No statistically significant differences in TC reduction were observed across subgroups of animal species, active component, or intervention duration. Heterogeneity was substantially lower in several subgroups, including the type 1 DN (I^2^ = 42.4%, *p* = 0.123), GRg1 (I^2^ = 13.4%, *p* = 0.315), medium-term intervention (I^2^ = 39.5%, *p* = 0.142), and mouse (I^2^ = 0.0%, *p* = 0.452) subgroups (Supplementary Table 5). A funnel plot (Figure 12B) and Egger’s test indicated statistically significant effects, suggesting publication bias for TC (*p* = 0.003; Figure 12C). Trim-and-fill analysis estimated that imputing two theoretically missing studies would yield a symmetric funnel plot (Figure 12D; Supplementary Table 3).

###### TG

**Figure 13:**
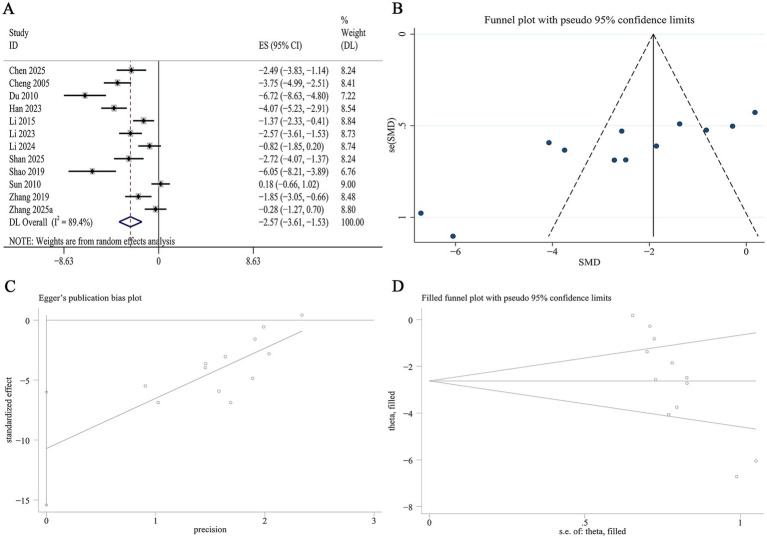
Effect of Panax notoginseng on TG. **(A)** Forest plot. **(B)** Funnel plot. **(C)** Egger’s publication bias plot. **(D)** Filled funnel plot.

12 studies reported data on TG levels. Meta-analysis demonstrated that Panax notoginseng significantly reduced TG levels compared to the control group [SMD: −2.57, 95% CI (−3.61, −1.53), *p* = 0.001; I^2^ = 89.4%, *p* = 0.000; Figure 13A]. Subgroup analyses stratified by animal species, DN model type, active component, and intervention duration revealed no statistically significant differences. Notably, heterogeneity was substantially lower in the NGR1 subgroup (I^2^ = 39.0%, *p* = 0.200; Supplementary Table 5). A funnel plot (Figure 13B) and Egger’s test indicated statistically significant effects, suggesting publication bias for TG outcomes (*p* = 0.000; Figure 13C). Trim-and-fill analysis was subsequently performed to adjust for this potential bias. Following imputation, the adjusted pooled effect size remained virtually unchanged, affirming the robustness of the finding for TG (Figure 13D).

##### Fibrosis biomarkers

###### TGF-β1

Four studies quantified TGF-β1 protein expression via immunohistochemistry, and six studies assessed its gene expression using RT-qPCR. Meta-analysis demonstrated that Panax notoginseng significantly reduced renal TGF-β1 expression compared to control at both the protein [SMD: −3.80, 95% CI (−6.10, −1.49), *p* = 0.037; I^2^ = 89.4%, *p* = 0.000] and gene level [SMD: −5.35, 95% CI (−7.86, −2.83), *p* = 0.013; I^2^ = 93.8%, *p* = 0.000]. Forest plots are provided in Supplementary Figure 2.

### Sensitivity analysis

A sensitivity analysis was conducted for the primary outcome measures: FBG, SCr, BUN, 24 h UPro, and KI. The results indicated that the pooled effect sizes remained stable and statistically significant, with no single study exerting a disproportionate influence. The detailed results are presented in Supplementary Figure 3.

### Dose-time-response relationship analysis

**Figure 14:**
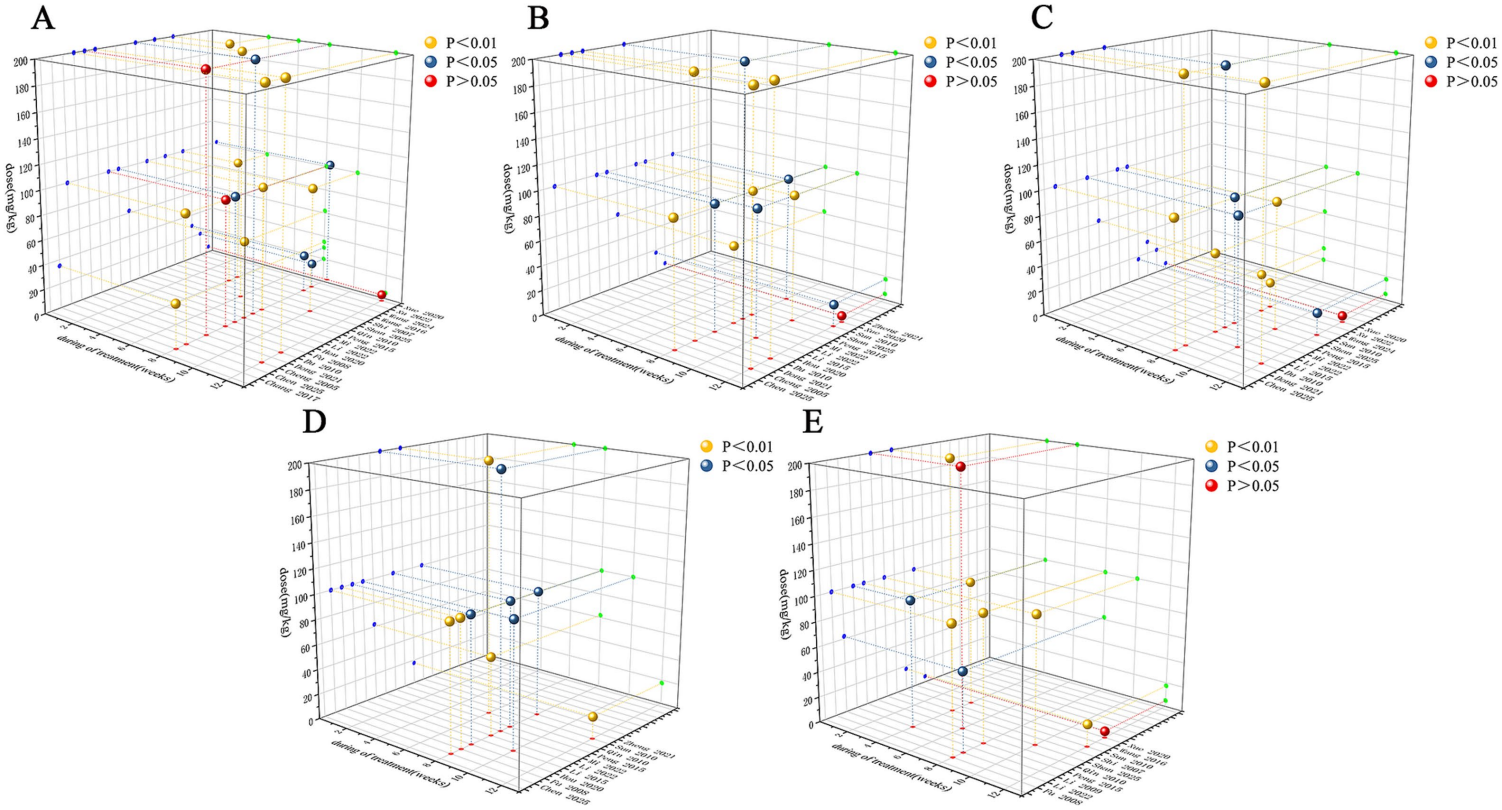
3-dimensional dose-time-response surface plots for **(A)** FBG, **(B)** SCr, **(C)** BUN, **(D)** 24 h UPro, and **(E)** KI.

To evaluate the combined influence of dosage and treatment duration on therapeutic efficacy, three-dimensional dose-time-response surface plots were constructed specifically for PNS. Due to the limited number of studies available for the other individual components (NGR1, GRg1, and GRb1), a reliable dose-time-response analysis could not be performed for them without introducing substantial bias. The analysis for PNS indicated that a dosage range of 20–200 mg/kg/d combined with a treatment duration of 4–12 weeks was associated with improvements in FBG (Figure 14A). For SCr (Figure 14B) and BUN (Figure 14C), the effective ranges were 17.5–200 mg/kg/d over 8–12 weeks. The effective duration for reducing 24 h UPro was 6–12 weeks (Figure 14D) and for lowering the KI it was 4–12 weeks (Figure 14E), within the same dosage range. In summary, the dose-time-response analysis suggested that a PNS regimen of 20–200 mg/kg/d administered for 8–12 weeks conferred optimal therapeutic benefits across multiple renal parameters in this preclinical model.

## Discussion

### Summary of evidence

This study included a total of 37 studies. The included animal studies exhibited diversity across several methodological dimensions, including species selection, disease modeling methods, specific Panax notoginseng components, and intervention durations. Notably, all studies exclusively utilized male animals, which may introduce a sex-related bias and limit the generalizability of the findings. Current scientific consensus advocates for the inclusion of both sexes in preclinical research to enhance the translational relevance and reliability of findings for subsequent clinical studies. Regarding methodological quality, it is recommended that future studies improve reporting rigor by detailing sequence generation, allocation concealment, and baseline characteristics, ensuring complete data reporting and proactively minimizing potential sources of bias.

The meta-analysis demonstrated that Panax notoginseng confers significant renoprotection in DN models, as evidenced by improvements in FBG, SCr, BUN, 24 h UPro, and KI. Treatment with Panax notoginseng effectively reduced serum levels of key inflammatory cytokines, including IL-1β, IL-6, TNF-*α*, and MCP-1. Regarding oxidative stress markers, Panax notoginseng treatment increased the levels of SOD and decreased the concentration of MDA in both serum and renal cortical tissue. In terms of anti-fibrotic effects, Panax notoginseng downregulated the expression of TGF-β1 at both the protein and gene levels. Furthermore, Panax notoginseng administration lowered serum concentrations of TC and TG. Collectively, these findings indicate that Panax notoginseng exerts multi-faceted therapeutic effects, encompassing anti-inflammatory, antioxidant, anti-fibrotic, and glucolipid metabolism-modulating activities.

Substantial heterogeneity was observed across the meta-analyses of primary outcomes. Subgroup analyses suggested that intervention duration may be a source of heterogeneity for SCr, the specific Panax notoginseng component may contribute to heterogeneity for 24 h UPro, and the DN model type, active component, and intervention duration may all be sources of heterogeneity for KI. Nevertheless, the precise reasons for the heterogeneity remain incompletely understood. It is also important to note that, while commercially available Panax notoginseng component products comply with pharmacopeial standards, few studies have employed multiple analytical methods to comprehensively characterize their chemical composition. Variability in the chemical composition of Panax notoginseng component may constitute an important source of the observed heterogeneity. This highlights the necessity for standardized and transparent reporting in future animal and clinical studies. Egger’s test indicated the presence of effects, suggestive of publication bias, for all primary outcomes. Following trim-and-fill adjustment, the pooled effect sizes for SCr and KI remained highly consistent with the unadjusted estimates. For FBG, BUN, and 24 h UPro, the adjusted effect sizes exhibited minor changes that did not alter the statistical significance of the results, further underscoring the robustness of the findings. The dose-time-response analysis demonstrated that a PNS regimen of 20–200 mg/kg/d administered for 8–12 weeks was associated with favorable therapeutic outcomes.

This study provides a comprehensive analysis of the efficacy of Panax notoginseng in DN, offering preclinical evidence to inform future clinical research and potential therapeutic development. It is important to acknowledge that the included studies often lacked in-depth investigation into the specific molecular mechanisms underlying the effects of Panax notoginseng on DN. Therefore, further high-quality preclinical studies are warranted to elucidate these mechanisms, thereby strengthening the translational foundation for Panax notoginseng in DN treatment.

### Possible protective mechanisms of Panax notoginseng

#### Anti-inflammatory effects

Immune-mediated inflammation is recognized as a key driver in the progression of DN (18). In the diabetic milieu, sustained hyperglycemia, accumulation of AGEs, oxidative stress, and other insults activate diverse signaling pathways. This leads to the upregulation of pro-inflammatory genes and the subsequent release of cytokines, including IL-1β, IL-6, TNF-*α*, and MCP-1. This persistent low-grade inflammatory state ultimately contributes to renal structural remodeling and the development of tubulointerstitial fibrosis (18–20). Multiple preclinical studies (21–29) have demonstrated that intervention with Panax notoginseng reduces the renal expression of pro-inflammatory factors, including IL-1β, IL-6, TNF-α, and MCP-1, in rodent models of DN, confirming its anti-inflammatory properties. For instance, Hou et al. (24) reported that PNS reduced macrophage infiltration in renal tissue. This anti-inflammatory effect may be mediated through inhibition of the nuclear factor kappa B (NF-κB) signaling pathway (29). Furthermore, NGR1, a constituent of Panax notoginseng, has been shown to reduce IL-1β and TNF-α expression, conferring protection to podocytes. This protective effect is potentially achieved by activating the phosphatidylinositol 3-kinase (PI3K)/protein kinase B (Akt) pathway and subsequently downregulating NF-κB expression (27). Similarly, GRg1 from Panax notoginseng suppresses the expression of multiple pro-inflammatory factors in DN models (22, 25, 26, 28, 30). This aligns with the conclusions of a prior meta-analysis, which reported that GRg1 alleviates inflammatory responses induced by high glucose (14). Mechanistic investigations further revealed that GRg1 increases the phosphorylation of PI3K and Akt and promotes the nuclear-to-cytoplasmic translocation of the transcription factor Forkhead box O3 (FOXO3) in DN models, events associated with the mitigation of renal inflammation and apoptosis (31). In summary, the anti-inflammatory action of Panax notoginseng in DN appears to be closely associated with the modulation of the PI3K/Akt and NF-κB signaling pathways. However, it should be noted that not all studies have consistently demonstrated these pathway-specific effects. For instance, while the PI3K/Akt pathway is frequently implicated, Chen et al. (32) suggest that its modulation by PNS may primarily manifest through the regulation of autophagy levels rather than direct inflammatory signaling. Additionally, others have reported that the anti-inflammatory effect of PNS, particularly the reduction of NF-κB p65 acetylation, may be mediated by the upregulation of silent information regulator 1 (SIRT1) (33). These divergent findings indicate that the mechanisms underlying the anti-inflammatory effects of Panax notoginseng in diabetic nephropathy are complex and likely involve multiple interacting pathways, warranting further in-depth investigation.

#### Anti-oxidative stress effects

In diabetes, hyperglycemia triggers excessive production of reactive oxygen species (ROS), disrupting the redox balance between ROS generation and the antioxidant defense (AOD) system (34, 35). This oxidative imbalance is a critical accelerator of DN onset and progression (34). The AOD system encompasses various antioxidant enzymes, including SOD and GSH-Px. The levels of these enzymes serve as key biomarkers of systemic and tissue-specific oxidative stress (36). Preclinical studies have shown that Panax notoginseng intervention in DN models significantly enhances the levels of renal antioxidant enzymes, including SOD and GSH-Px, while reducing levels of lipid peroxidation end-products such as MDA (23, 29, 37–46). These findings suggest that the effects of Panax notoginseng in DN are attributable to its potent antioxidant properties. Nuclear factor erythroid 2-related factor 2 (Nrf2) is a master transcription factor that orchestrates the cellular antioxidant response. Upon activation, it binds to the antioxidant response element in target gene regions, initiates transcription, and promotes the expression of various antioxidant genes (47). Mechanistic studies have demonstrated that PNS dose-dependently activates the renal Nrf2 pathway in DN mice. This activation upregulates the expression of downstream effector heme oxygenase-1 (HO-1), leading to increased SOD activity and decreased MDA levels (29, 46). Furthermore, NGR1 exhibits potent *in vivo* antioxidant activity. Intervention with NGR1 in rodent models elevates SOD and GSH-Px activities and reduces MDA content in both serum and renal tissue (48). NGR1 also upregulates Nrf2-mediated HO-1 expression in db/db mice. Complementary *in vitro* experiments showed that NGR1 inhibits AGE-induced ROS generation in human renal proximal tubular cells, consequently attenuating apoptosis (49, 50). Similarly, GRg1 demonstrates significant antioxidant capacity. It restores the activity of depleted antioxidant enzymes (14, 25, 28, 30, 51) and mitigates oxidative stress through modulation of the Nrf2/HO-1 signaling axis (52). Collectively, the antioxidant efficacy of Panax notoginseng in DN treatment is linked to the regulation of the Nrf2 signaling pathway.

#### Regulation of glycolipid metabolism

Glucolipid metabolic disorders (GLMDs), characterized by dysregulation in the synthesis, catabolism, and absorption of glucose and lipids, constitute both a hallmark of DN and a direct contributor to its pathogenesis (53). Numerous studies have demonstrated the hypoglycemic effect of Panax notoginseng, showing it significantly improves FBG levels in DN animal models (21–24, 26–30, 32, 37–41, 46, 48–51, 54–63). Furthermore, it has been shown to significantly lower serum levels of TC and TG (21, 22, 30, 32, 37, 42, 44, 49, 57, 60, 63). Supporting these preclinical findings, a meta-analysis of RCTs indicated that Xuesaitong Injection—whose primary active component is PNS—combined with conventional therapy, significantly improved glucolipid profiles in patients with diabetes (64). This affirms the translational potential and clinical efficacy of Panax notoginseng in managing GLMDs. Notably, the amelioration of GLMDs by PNS, NGR1, and GRg1 likely underpins their broader therapeutic benefits, including anti-inflammatory, antioxidant, and anti-fibrotic effects, given the interconnected nature of metabolic and pathological pathways in DN. Furthermore, Han et al. (60) demonstrated that high-fat diet-induced abnormal lipid metabolism and consequent renal lipid deposition are key drivers of renal damage and fibrosis progression in a T2DM mouse model. In this context, NGR1 was found to attenuate renal lipid deposition and pathological injury in diabetic mice by reducing CD36 overexpression and suppressing the transient receptor potential cation channel 6 (TRPC6)/nuclear factor of activated T-cells 2 (NFAT2) signaling pathway (60). However, the precise molecular mechanisms by which Panax notoginseng exert their glucolipid-modulating effects remain incompletely understood and warrant further in-depth investigation.

#### Anti-fibrosis effects

Renal fibrosis is a pivotal pathological hallmark and central pathogenic mechanism in DN, primarily driven by excessive extracellular matrix (ECM) accumulation, epithelial-mesenchymal transition (EMT) (65). TGF-*β* is the most extensively characterized profibrotic cytokine. Under hyperglycemic conditions, TGF-β1 transcription is upregulated in various renal cell types, leading to activation of the small mothers against decapentaplegic (Smad) signaling pathway and consequent acceleration of renal fibrogenesis (65, 66). Within the canonical TGF-β/Smad pathway, individual Smad proteins serve distinct functions: Smad2 and Smad3 act as major downstream transcriptional mediators of fibrosis, whereas Smad7 functions as a critical negative regulator by competitively binding TGF-β receptors, promoting receptor degradation, and inhibiting Smad2/3 phosphorylation (66). Multiple studies have demonstrated that PNS intervention in DN models significantly reduces renal TGF-β1 levels and the expression of fibrosis-related ECM proteins (24, 38, 41, 44, 55–57). These findings suggest that the anti-fibrotic effect of PNS is mediated through suppression of the TGF-β1 signaling axis. Further mechanistic insights indicate that PNS modulates downstream TGF-β1 signaling by upregulating inhibitory Smad7 (56) and downregulating pro-fibrotic mitogen-activated protein kinase (MAPK) activity (44), thereby engaging multiple anti-fibrotic pathways. Similarly, NGR1 inhibits the TGF-β1 pathway and reduces renal collagen deposition in db/db mice. This anti-fibrotic action may be linked to its concurrent activation of the Nrf2/HO-1 pathway (49). Supporting this notion, *in vitro* experiments showed that the anti-fibrotic effect of NGR1 on HK-2 cells under AGE stimulation was abolished upon pharmacological inhibition of HO-1 (49). Du et al. (51) reported that GRg1 significantly downregulated renal expression of TGF-β1 and Smad2/3 while upregulating Smad7 in a rat model of DN. In a high-fat diet-induced C57BL/6 J mouse model of DN, Han et al. (60) further demonstrated that GRg1 inhibits the TGF-*β*1/Smad2/3 pathway, reduces cortical collagen IV (Col IV) expression, and alleviates both glomerular and tubulointerstitial fibrosis. In summary, although renal fibrosis involves highly complex mechanisms, current evidence strongly implicates the TGF-β signaling pathway as a central target through which Panax notoginseng exert their anti-fibrotic effects in DN.

#### Safety of Panax notoginseng

Panax notoginseng is generally regarded as having a favorable safety profile. Preparations primarily composed of PNS, such as Xuesaitong capsules and injections, have been clinically utilized in China for over four decades (67). A real-world study (68) reported that the incidences of adverse drug reactions (ADRs) and adverse drug events (ADEs) following Xuesaitong injection were 0.33 and 0.5%, respectively. Common manifestations included skin erythema, pruritus, foot pain, and gastrointestinal discomfort. Importantly, the occurrence of these ADRs did not necessitate treatment discontinuation or exacerbate underlying conditions, further supporting its acceptable safety profile in clinical practice. Similarly, a meta-analysis focusing on Xuesaitong soft capsules (69) also documented a low incidence of ADRs. Collectively, clinical studies have demonstrated a favorable safety profile for Panax notoginseng. However, it is noteworthy that most of these trials involved relatively short observation periods. Therefore, long-term safety surveillance in future studies remains warranted.

Regarding the preclinical toxicity of Panax notoginseng, the existing data are less comprehensive. Wang et al. (69) identified developmental toxicity associated with a Panax notoginseng decoction extract in a zebrafish model. The proposed mechanism involves the downregulation of carboxypeptidase A1 (CPA1) and opioid growth factor receptor-like 2 (OGFRL2). These findings suggest potential risks associated with the use of Panax notoginseng in pediatric populations. Studies on PNS (70) reported a median lethal dose (LD₅₀) in mice of 6784.2 mg·kg^−1^, approximately 130 times the typical clinical dose for adults. Acute toxicity studies revealed no significant toxic reactions. However, long-term, high-dose administration has been associated with hepatic and gastrointestinal damage in animal models. Nonetheless, at clinically recommended doses, its use is considered to have an acceptable safety margin.

### Limitations

Despite adherence to the PRISMA guidelines, this study has several limitations: (1) Although Panax notoginseng has been shown to have therapeutic effects on DN, differences exist between animal models and human DN pathology. Therefore, the translational significance of these preclinical findings for human patients still needs to be validated through future clinical research. The original studies included in this analysis generally provided insufficient chemical characterization of Panax notoginseng and lacked standardized reporting in accordance with ConPhyMP statement. This deficiency may compromise the reliability and reproducibility of the findings; (2) Substantial heterogeneity was observed for the primary outcomes. Although subgroup analyses were performed to explore potential sources, they could not be fully explained, introducing uncertainty into the corresponding pooled estimates; (3) A considerable proportion of the included studies originated from non-indexed journals or Chinese university databases. The peer-review standards of these publications may differ from those of mainstream indexed journals, potentially introducing bias and affecting the reliability of the original data. Although sensitivity analysis indicated that the conclusions were not significantly affected, this factor remains a source of potential heterogeneity and limits the overall strength of the evidence. Future research should prioritize high-quality, rigorously peer-reviewed studies to validate these findings; (4) Despite the application of random-effects models, methodological heterogeneity arising from variations in the measurement techniques for the same outcome across studies may have introduced systematic error into the meta-analysis; (5) Evidence of publication bias was detected for primary outcomes. While trim-and-fill analysis suggested the pooled effect sizes were robust to this bias, an overestimation of the true treatment effect remains possible, as studies with positive results are more likely to be published; (6) A portion of the data was extracted from published figures using digitization software (WebPlotDigitizer 4.7), a process that may introduce minor measurement inaccuracies; and (7) Only data from the final time point of each study were included to maintain statistical independence. However, this approach ignores the temporal dynamics of treatment effects and may introduce bias, as studies with different durations capture different stages of treatment response.

## Conclusion

This systematic review and meta-analysis demonstrates that Panax notoginseng exerts renoprotective effects in animal models of DN. The underlying mechanisms likely involve anti-inflammatory, antioxidant, glucolipid metabolism-regulating, and anti-fibrotic activities. Our novel exploration of the dose-time-response relationship for PNS in DN treatment indicates that an effective regimen is 20–200 mg/kg/d administered for 8–12 weeks. Despite the heterogeneity observed in some analyses, this study robustly supports the therapeutic potential of Panax notoginseng for DN. It provides a preclinical evidence base to inform the design of future high-quality research and potential clinical translation.

## Data Availability

The original contributions presented in the study are included in the article/Supplementary material, further inquiries can be directed to the corresponding author.

## Glossary

ADEs: adverse events
ADRs: adverse reactions
AGEs: advanced glycation end products
AOD: antioxidant defense
BUN: blood urea nitrogen
DM: diabetes mellitus
DN: diabetic nephropathy
ECM: extracellular matrix
FBG: fasting blood glucose
GLMDs: glycolipid metabolism disorders
GSH-Px: glutathione peroxidases
GS-Rb1: ginsenoside Rb1
GS-Rg1: ginsenoside Rg1
HO-1: heme oxygenase 1
IL-1β: interleukin 1β
IL-6: interleukin 6
KI: kidney index
MAPK: mitogen-activated protein kinase
MCP-1: monocyte chemotactic protein 1
MDA: malondialdehyde
NF-κB: nuclear factor κB
Nrf2: nuclear factor erythroid 2-related factor 2
NGR1: notoginsenoside R1
PNS: panax notoginseng saponins
ROS: reactive oxygen species
Smad: small mother against decapentaplegic
SCr: serum creatinine
SOD: superoxide dismutase
TC: total cholesterol
TG: triglyceride
TGF-*β*: transforming growth factor β
TNF-*α*: tumor necrosis factor α
24 h UPro: 24-h urine protein
