# Supplementary material for: The effects of Panax notoginseng for the treatment of diabetic nephropathy in animals: a systematic review and meta-analysis
Source: Front Nutr. 2026 Mar 17;13:1780933. doi: 10.3389/fnut.2026.1780933 (PMC13036193; doi:10.3389/fnut.2026.1780933)
Supplement: Supplementary file 1 [file Data_Sheet_1.PDF]

Supplementary Table 1. Search strategy of each database.

Databases	Search strategies
**PubMed**	(("Diabetic Nephropathies"[Mesh]) OR ((((((((((((((((Nephropathies, Diabetic[Title/Abstract]) OR (Nephropathy, Diabetic[Title/Abstract])) OR (Diabetic Kidney Disease[Title/Abstract])) OR (Diabetic Kidney Diseases[Title/Abstract])) OR (Kidney Disease, Diabetic[Title/Abstract])) OR (Kidney Diseases, Diabetic[Title/Abstract])) OR (Diabetic Nephropathy[Title/Abstract])) OR (Diabetic Glomerulosclerosis[Title/Abstract])) OR (Glomerulosclerosis, Diabetic[Title/Abstract])) OR (Intracapillary Glomerulosclerosis[Title/Abstract])) OR (Kimmelstiel-Wilson Disease[Title/Abstract])) OR (Kimmelstiel Wilson Disease[Title/Abstract])) OR (Nodular Glomerulosclerosis[Title/Abstract])) OR (Glomerulosclerosis, Nodular[Title/Abstract])) OR (Kimmelstiel-Wilson Syndrome[Title/Abstract])) OR (Kimmelstiel Wilson Syndrome[Title/Abstract])) OR (Syndrome, Kimmelstiel-Wilson[Title/Abstract]))) AND (("Panax notoginseng"[Mesh]) OR ((((((((((((notoginsengs, Panax[Title/Abstract])) OR (Panax notoginsengs[Title/Abstract])) OR (sanqi[Title/Abstract])) OR (xuesetong[Title/Abstract])) OR (xue sai tong[Title/Abstract])) OR (xuesaitong[Title/Abstract])) OR (xuesetong injection[Title/Abstract])) OR (Panax notoginseng extract[Title/Abstract])) OR (Xue shuan tong[Title/Abstract])) OR (Xueshuantong[Title/Abstract])) OR (ginsenoside Rb1[Title/Abstract])) OR (ginsenoside Rg1[Title/Abstract])) OR (Notoginsenoside[Title/Abstract])))
**Web of science**	#3 #1 AND #2 and Preprint Citation Index (Exclude – Database)<br>#2 Panax notoginseng (Topic) or notoginsengs, Panax (Topic) or Panax notoginsengs (Topic) or sanqi (Topic) or xuesetong (Topic) or xue sai tong (Topic) or xuesaitong (Topic) or xuesetong injection (Topic) or Panax notoginseng extract (Topic) or Xue shuan tong (Topic) or Xueshuantong (Topic) or ginsenoside Rb1 (Topic) or ginsenoside Rg1 (Topic) or Notoginsenoside (Topic) and Preprint Citation Index (Exclude – Database) and Research Commons (Exclude – Database)<br>#1 Diabetic Nephropathies (Topic) or Nephropathies, Diabetic (Topic) or Nephropathy, Diabetic (Topic) or Diabetic Kidney Disease (Topic) or Diabetic Kidney Diseases (Topic) or Kidney Disease, Diabetic (Topic) or Kidney Diseases, Diabetic (Topic) or Diabetic Nephropathy (Topic) or Diabetic Glomerulosclerosis (Topic) or Glomerulosclerosis, Diabetic (Topic) or Intracapillary Glomerulosclerosis (Topic) or Kimmelstiel-Wilson Disease (Topic) or Kimmelstiel Wilson Disease (Topic) or Nodular Glomerulosclerosis (Topic) or Glomerulosclerosis, Nodular (Topic) or Kimmelstiel-Wilson Syndrome (Topic) or Kimmelstiel Wilson Syndrome (Topic) or Syndrome, Kimmelstiel-Wilson (Topic) and Preprint Citation Index (Exclude – Database) and Research Commons (Exclude – Database)
**Embase**	#7 #3 AND #6<br>#6 #4 OR #5<br>#5 'notoginsengs, Panax':ab,ti OR 'Panax notoginsengs':ab,ti OR 'sanqi':ab,ti OR 'xuesetong':ab,ti OR 'xue sai tong':ab,ti OR 'xuesaitong':ab,ti OR 'xuesetong

	injection':ab,ti OR 'Panax notoginseng extract':ab,ti OR 'Xue shuan tong':ab,ti OR 'Xueshuantong':ab,ti OR 'ginsenoside Rb1':ab,ti OR 'ginsenoside Rg1':ab,ti OR 'Notoginsenoside':ab,ti<br>#4 'Panax notoginseng'/exp<br>#3 #1 OR #2<br>#2 'nephropathies, diabetic':ab,ti OR 'nephropathy, diabetic':ab,ti OR 'diabetic kidney disease':ab,ti OR 'diabetic kidney diseases':ab,ti OR 'kidney disease, diabetic':ab,ti OR 'kidney diseases, diabetic':ab,ti OR 'diabetic glomerulosclerosis':ab,ti OR 'glomerulosclerosis, diabetic':ab,ti OR 'intracapillary glomerulosclerosis':ab,ti OR 'kimmelstiel-wilson disease':ab,ti OR 'kimmelstiel wilson disease':ab,ti OR 'nodular glomerulosclerosis':ab,ti OR 'glomerulosclerosis, nodular':ab,ti OR 'kimmelstiel-wilson syndrome':ab,ti OR 'kimmelstiel wilson syndrome':ab,ti OR 'syndrome, kimmelstiel-wilson':ab,ti<br>#1 'diabetic nephropathy'/exp
**CBM**	7 (#6) OR (#5)<br>6 (#4) OR (#3)<br>5 (#2) OR (#1)<br>4 "参三七"[常用字段:智能] OR "田三七"[常用字段:智能] OR "田七"[常用字段:智能] OR "三七皂苷"[常用字段:智能] OR "三七皂甙"[常用字段:智能] OR "人参皂苷Rb1"[常用字段:智能] OR "人参皂苷Rg1"[常用字段:智能] OR "血塞通"[常用字段:智能] OR "血栓通"[常用字段:智能]<br>3 "三七"[不加权:扩展]<br>2 "毛细管间性肾小球硬化症"[常用字段:智能] OR "糖尿病性肾小球硬化症"[常用字段:智能] OR "Kimmelstiel"[常用字段:智能] AND "Wilson 病"[常用字段:智能] OR "结节性肾小球硬化症"[常用字段:智能] OR "糖尿病肾疾病"[常用字段:智能] OR "基-威综合征"[常用字段:智能] OR "Kimmelstiel-Wilson 病"[常用字段:智能] OR "糖尿病肾脏病"[常用字段:智能] OR "糖尿病肾脏疾病"[常用字段:智能] OR "糖尿病性肾病"[常用字段:智能] OR "糖尿病性肾小球硬化"[常用字段:智能]<br>1 "糖尿病肾病"[不加权:扩展]
**CNKI**	SU%=('糖尿病肾病' + '糖尿病肾脏病' + '糖尿病肾脏疾病' + '糖尿病性肾病' + '糖尿病性肾小球硬化') * ('三七' + '三七皂苷' + '人参皂苷 Rb1' + '人参皂苷 Rg1' + '血塞通' + '血栓通')
**WanFang**	主题: (("糖尿病肾病" or "糖尿病肾脏病" or "糖尿病肾脏疾病" or "糖尿病性肾病" or "糖尿病性肾小球硬化") and ("三七" or "三七皂苷" or "人参皂苷 Rb1" or "人参皂苷 Rg1" or "血塞通" or "血栓通"))

**VIP**	M=((糖尿病肾病 OR 糖尿病肾脏病 OR 糖尿病肾脏疾病 OR 糖尿病性肾病 OR 糖尿病性肾小球硬化) AND (三七 OR 三七皂苷 OR 人参皂苷 Rb1 OR 人参皂苷 Rg1 OR 血塞通 OR 血栓通))

Supplementary Table 2. The batch numbers or purity on Panax notoginseng.

Author, year	Specific component	batch numbers or purity
Chang 2017	PNS	Shanghai Leading Biotechnology Co., Ltd.
Chen 2025	PNS	Dalian Meilun Biotechnology Co., Ltd. (MB6091)
Cheng 2005	PNS	Yunnan Jintaide Pharmaceutical Co., Ltd. (purity 98.4%)
Dong 2021	PNS	Shanghai Guangrui Biotechnology Co., Ltd. (GR-19070601, putiry 98%)
Du 2010	PNS	Yunnan Sanqi Pharmaceutical Co., Ltd.
Du 2018	GRg1	ChengDu ConBon Bio-tech Co., Ltd，purity ≥98%
Fu 2008	PNS	Heilongjiang Zhenbaodao Pharmaceutical Co., Ltd.
Gui 2014	NGR1	ShangHai YuanYe Biotechnology Co.,Ltd，＞98%
Han 2023	GRg1	Chengdu Desite Biotechnology Co., Ltd，>98%
Hou 2020	PNS	National Institutes for Food and Drug Control (NIFDC), China (120941.200807)
Huang 2016	NGR1	Sigma-Aldrich Chemicals, purity >98%
Li 2009	PNS	Kunming Pharmaceutical Group Co., Ltd. (Z20026438)
Li 2015	PNS	Beijing Tongrentang Pharmaceutical Co., Ltd.
Li 2018	GRg1	Shanghai Yansheng Corporation (purity 99.6%)
Li 2022	PNS	Xi'an Tianbao Biotechnology Co., Ltd. (TB20171210, purity 99%)
Li 2023	GRg1	Yunnan Tean'na Pharmaceutical Co., Ltd.
Li 2024	NGR1	Shaanxi Zhonghe Jiantai Bioengineering Co., Ltd.
Mi 2022	PNS	Sanjiu Medical & Pharmaceutical
Peng 2015	PNS	Guangxi Wuzhou Pharmaceutical (Group) Co., Ltd. (130628)
Qin 2010	PNS	Yunnan Jintaide Pharmaceutical Co., Ltd. (purity 95%)
Ruan 2021	GRg1	NR
Shan 2025	PNS	Chengdu Herbpurify Bio-technology Co., Ltd.
Shao 2019	GRb1	weikeqi-biotech, Sichuan,
Shi 2007	PNS	Yunnan Jintaide Pharmaceutical Co., Ltd. (0507305, purity 95%)
Sun 2010	PNS	广西梧州制药集团股份有限公司,批准文号,国药准字 20025652
Wang 2016	PNS	Harbin Zhenbao Pharmaceutical Co., Ltd. (20130511)
Wang 2024	PNS	Shanghai Yuanye Bio-Technology Co., Ltd. (B21102, purity ≥90.1%)
Xu 2012	PNS	Heilongjiang Zhenbaodao Pharmaceutical Co., Ltd.
Xu 2022	PNS	Shanghai Yuanye Bio-Technology Co., Ltd. (B21102)
Xue 2020	PNS	Yige Pharmaceutical Co. Ltd
Yang 2020	GRg1	Shanghai Slack Jingda Co., Ltd.
Zhang 2009	GRg1	Yunnan Plant Pharmaceutical Co., Ltd. (purity > 98.0％)
Zhang 2019	NGR1	Shanghai Winherb Medical S&T Development (purity > 98.6%)

Zhang 2023	NGR1	Chengdu Pinfeld Biotechnology Co., Ltd. (purity > 98%)
Zhang 2025a	GRg1	Xi'an Tiangongyuan Biotechnology Co., Ltd.
Zhang 2025b	GRg1	Chengdu Desite Biotechnology Co., Ltd. (purity ≥ 98%)
Zheng 2021	PNS	NR

Supplementary Table 3. Subgroup analysis of primary outcomes.

Variables	SMD [95%CI]	*P* value	I^2(%)	*P*-heterogeneity
**FBG**				
DN models				
type 1 DN	-3.16 [-4.23, -2.10]	0.000	92.9	0.000
type 2 DN	-2.46 [-3.41, -1.51]	0.000	87.1	0.000
specific drug				
PNS	-2.98 [-4.02, -1.94]	0.000	93.0	0.000
NR1	-1.51 [-2.35, -0.68]	0.000	69.6	0.011
Rg1	-3.53 [-5.49, -1.57]	0.000	90.5	0.000
Rb1	-5.35 [-7.30, -3.39]	0.000	-	-
duration				
<8 weeks	-4.18 [-6.42, -1.94]	0.000	89.7	0.000
8≤t < 12 weeks	-2.79 [-3.81, -1.77]	0.000	92.3	0.000
≥12 weeks	-2.44 [-3.61, -1.27]	0.000	88.7	0.000
species				
rats	-3.22 [-4.10, -2.35]	0.000	90.4	0.000
mice	-1.87 [-3.19, -0.56]	0.005	92.0	0.000
**SCr**				
DN models				
type 1 DN	-2.98 [-3.82, -2.15]	0.000	88.3	0.000
type 2 DN	-2.26 [-3.31, -1.20]	0.000	89.9	0.000
specific drug				
PNS	-2.77 [-3.60, -1.95]	0.000	84.5	0.000
NR1	-1.71 [-3.11, -0.31]	0.016	87.5	0.000
Rg1	-2.83 [-4.20, -1.45]	0.000	92.3	0.000
Rb1	-4.61 [-6.35, -2.87]	0.000	-	-
duration				
<8 weeks	-0.27 [-0.40, -0.95]	0.428	22.0	0.257
8≤t < 12 weeks	-3.12 [-3.92, -2.31]	0.000	85.0	0.000

≥12 weeks	-2.61 [-3.68, -1.55]	0.000	89.0	0.000
species				
rats	-2.61 [-3.39, -1.82]	0.000	90.1	0.000
mice	-2.93 [-4.08, -1.77]	0.000	84.1	0.000
**BUN**				
DN models				
type 1 DN	-2.09 [-2.86, -1.33]	0.000	86.2	0.000
type 2 DN	-3.18 [-4.31, -2.04]	0.000	90.6	0.000
specific drug				
PNS	-2.05 [-2.69, -1.41]	0.000	78.2	0.000
NR1	-1.60 [-2.63, -0.57]	0.002	79.3	0.001
Rg1	-4.13 [-6.02, -2.24]	0.000	93.8	0.000
duration				
<8 weeks	-2.24 [-5.83, -1.36]	0.222	95.5	0.000
8≤t < 12 weeks	-2.53 [-3.32, -1.75]	0.000	86.7	0.000
≥12 weeks	-2.78 [-4.14, -1.43]	0.000	91.5	0.000
species				
rats	-3.22 [-4.31, -2.12]	0.000	89.1	0.000
mice	-2.24 [-3.05, -1.43]	0.009	87.1	0.000
**24h Upro**				
DN models				
type 1 DN	-3.44 [-4.59, -2.28]	0.000	89.8	0.000
type 2 DN	-5.41 [-7.93, -2.90]	0.000	80.4	0.006
specific drug				
PNS	-3.78 [-5.16, -2.41]	0.000	91.5	0.000
NR1	-2.50 [-3.50, -1.49]	0.000	32.1	0.225
Rg1	-6.35 [-8.12, -4.58]	0.000	20.2	0.263
duration				
<8 weeks	-1.55 [-2.41, -0.69]	0.000	-	-
8≤t < 12 weeks	-5.18 [-6.96, -3.40]	0.000	92.7	0.000
≥12 weeks	-2.01 [-3.12, -0.90]	0.000	69.7	0.037
species				
rats	-3.44 [-4.59, -2.28]	0.000	89.8	0.000
mice	-5.41 [-7.93, -2.90]	0.000	80.4	0.006
**KI**				
DN models				

type 1 DN	-1.82 [-2.16, -1.47]	0.000	25.0	0.198
type 2 DN	-1.80 [-4.14, -0.53]	0.129	93.0	0.000
specific drug				
PNS	-1.76 [-2.14, -1.37]	0.000	25.5	0.209
NR1	-1.06 [-2.50, -0.38]	0.149	80.2	0.025
Rg1	-2.58 [-6.01, -0.85]	0.141	94.6	0.000
Rb1	-2.78 [-4.04, -1.52]	0.000	-	-
duration				
<12 weeks	-1.94 [-3.04, -0.84]	0.001	85.6	0.000
8≤t < 12 weeks	-1.52 [-1.98, -1.06]	0.000	0.0	0.478
≥12 weeks	-2.14 [-2.67, -1.61]	0.000	0.0	0.551

DN: diabetic nephropathy; FBG: fasting blood glucose; BUN: blood urea nitrogen; SCr: serum creatinine; 24h Upro: 24-hour urine protein; KI: kidney index; SMD: standardized mean differences

Supplementary Table 4. Results from trim-and-fill analysis.

**Variables**	**No. of Trials**	**SMD [95%CI]**	***P* value**
**FBG**			
before trim and fill	29	-3.17 [-4.11, -2.23]	0.000
after trim and fill	35	-4.10 [-5.22, -2.98]	0.000
**BUN**			
before trim and fill	25	-2.67 [-3.43, -1.90]	0.000
after trim and fill	27	-2.90 [-3.68, -2.11]	0.000
**24h Upro**			
before trim and fill	14	-4.45 [-6.02, -2.87]	0.000
after trim and fill	16	-5.08 [-6.82, -3.34]	0.000
**Serum MDA**			
before trim and fill	12	-3.74 [-5.05, -2.43]	0.000
after trim and fill	14	-4.33 [-5.75, -2.91]	0.000
**TC**			
before trim and fill	11	-2.37 [-3.26, -1.48]	0.000
after trim and fill	13	-2.79 [-3.73, -1.84]	0.000

FBG: fasting blood glucose; KI: kidney index; SMD: standardized mean differences

Supplementary Table 5. Subgroup analysis of Oxidative Stress Biomarkers.

**Variables**	**SMD [95%CI]**	***P* value**	**I^2(%)**	***P*-heterogeneity**

**Serum** SOD				
DN models				
type 1 DN	4.03 [2.71, 5.35]	0.000	84.6	0.000
type 2 DN	3.16 [-2.38, 8.70]	0.263	95.4	0.000
specific drug				
PNS	4.24 [2.27, 6.20]	0.000	86.1	0.000
NR1	2.52 [1.46, 3.58]	0.000	-	-
Rg1	4.10 [-0.10, 8.31]	0.056	95.9	0.000
Rb1	3.45 [2.02, 4.87]	0.000	-	-
duration				
<8 weeks	2.65 [1.31, 4.00]	0.000	-	-
8≤t < 12 weeks	2.36 [0.78, 3.93]	0.003	82.8	0.001
≥12 weeks	5.46 [3.26, 7.66]	0.000	88.4	0.000
species				
rats	4.25 [2.97, 5.54]	0.000	84.6	0.000
mice	0.42 [-0.57, 1.41]	0.407	-	-
**Renal SOD**				
DN models				
type 1 DN	2.08 [1.25, 2.91]	0.000	80.0	0.000
type 2 DN	2.56 [1.59, 3.53]	0.000	-	-
specific drug				
PNS	2.05 [1.16, 2.94]	0.000	80.9	0.000
NR1	3.24 [2.04, 4.44]	0.000	-	-
Rb1	1.74 [0.69, 2.78]	0.001	-	-
duration				
<8 weeks	2.22 [0.98, 3.47]	0.000	-	-
8≤t < 12 weeks	2.05 [1.06, 3.05]	0.000	83.2	0.000
≥12 weeks	2.46 [0.99, 3.93]	0.001	70.6	0.065
species				
rats	2.12 [1.30, 2.95]	0.000	82.5	0.000
mice	2.18 [1.21, 3.15]	0.000	34.8	0.215
**Serum MDA**				
DN models				
type 1 DN	-3.42 [-4.49, -2.36]	0.000	83.3	0.000
type 2 DN	-3.13 [-7.29, 1.04]	0.141	82.9	0.000

specific drug				
PNS	-2.91 [-4.11, -1.71]	0.000	81.1	0.000
NR1	-2.82 [-3.94, -1.70]	0.000	-	-
Rg1	-3.47 [-6.03, -0.91]	0.008	91.2	0.000
Rb1	-7.67 [-10.32, -5.01]	0.000	-	-
duration				
<8 weeks	-7.32 [-16.38, 1.74]	0.113	94.4	0.000
8≤t < 12 weeks	-1.88 [-2.61, -1.14]	0.000	36.9	0.191
≥12 weeks	-3.83 [-5.29, -2.38]	0.000	84.8	0.000
species				
rats	-3.60 [-4.64, -2.55]	0.000	83.4	0.000
mice	-1.08 [-2.14, -0.02]	0.045	-	-
**Renal MDA**				
DN models				
type 1 DN	-2.93 [-3.96, -1.90]	0.000	83.6	0.000
type 2 DN	-2.05 [-3.80, -0.29]	0.022	86.8	0.006
specific drug				
PNS	-2.30 [-3.09, -1.51]	0.000	78.7	0.000
NR1	-3.64 [-4.93, -2.35]	0.000	-	-
Rb1	-6.31 [-8.54, -4.07]	0.000	-	-
duration				
<8 weeks	-3.66 [-5.26, -2.06]	0.000	-	-
8≤t < 12 weeks	-2.15 [-2.96, -1.35]	0.000	78.6	0.000
≥12 weeks	-4.81 [-7.40, -2.22]	0.000	75.6	0.043
species				
rats	-2.95 [-4.12, -1.78]	0.000	85.1	0.000
mice	-2.32 [-3.60, -1.04]	0.000	81.5	0.005

DN: diabetic nephropathy; FBG: fasting blood glucose; BUN: blood urea nitrogen; SCr: serum creatinine; 24h Upro: 24-hour urine protein; KI: kidney index; SMD: standardized mean differences

Supplementary Table 6. Subgroup analysis of Glucose and Lipid Metabolism Markers.

Variables	SMD [95%CI]	*P* value	I^2(%)	*P*-heterogeneity
TC				
DN models				
type 1 DN	-3.46 [-4.98, -1.94]	0.000	84.6	0.000

type 2 DN	-1.45 [-2.00, -0.90]	0.000	42.4	0.123
specific drug				
PNS	-2.79 [-3.94, -1.64]	0.000	77.3	0.001
NR1	-1.32 [-2.92, 0.29]	0.000	74.8	0.047
Rg1	-1.40 [-1.96, 0.85]	0.109	13.4	0.315
Rb1	-6.01 [-8.15, -3.86]	0.000	-	-
duration				
<8 weeks	-0.54 [-1.54, 0.46]	0.288	-	-
8≤t < 12 weeks	-1.80 [-2.36, -1.25]	0.000	39.5	0.142
≥12 weeks	-3.70 [-5.76, -1.64]	0.000	88.7	0.000
species				
rats	-2.44 [-3.50, -1.38]	0.000	85.3	0.000
mice	-1.87 [-2.47, -1.27]	0.000	0.0	0.452
**TG**				
DN models				
type 1 DN	-3.04 [-4.85, -1.23]	0.001	92.5	0.000
type 2 DN	-2.18 [-3.46, -0.91]	0.001	86.5	0.000
specific drug				
PNS	-2.70 [-4.39, -1.02]	0.002	91.6	0.000
NR1	-1.29 [-2.30, -0.29]	0.012	39.0	0.200
Rg1	-2.29 [-4.45, -0.13]	0.038	92.0	0.000
Rb1	-6.05 [-8.21, -3.89]	0.000	-	-
duration				
<8 weeks	-0.82 [-1.85, 0.20]	0.116	-	-
8≤t < 12 weeks	-2.22 [-3.31, -1.13]	0.000	82.3	0.000
≥12 weeks	-3.53 [-6.02, -1.04]	0.005	94.3	0.000
species				
rats	-2.51 [-3.79, -1.23]	0.000	90.9	0.000
mice	-2.82 [-4.17, -1.47]	0.000	72.2	0.028

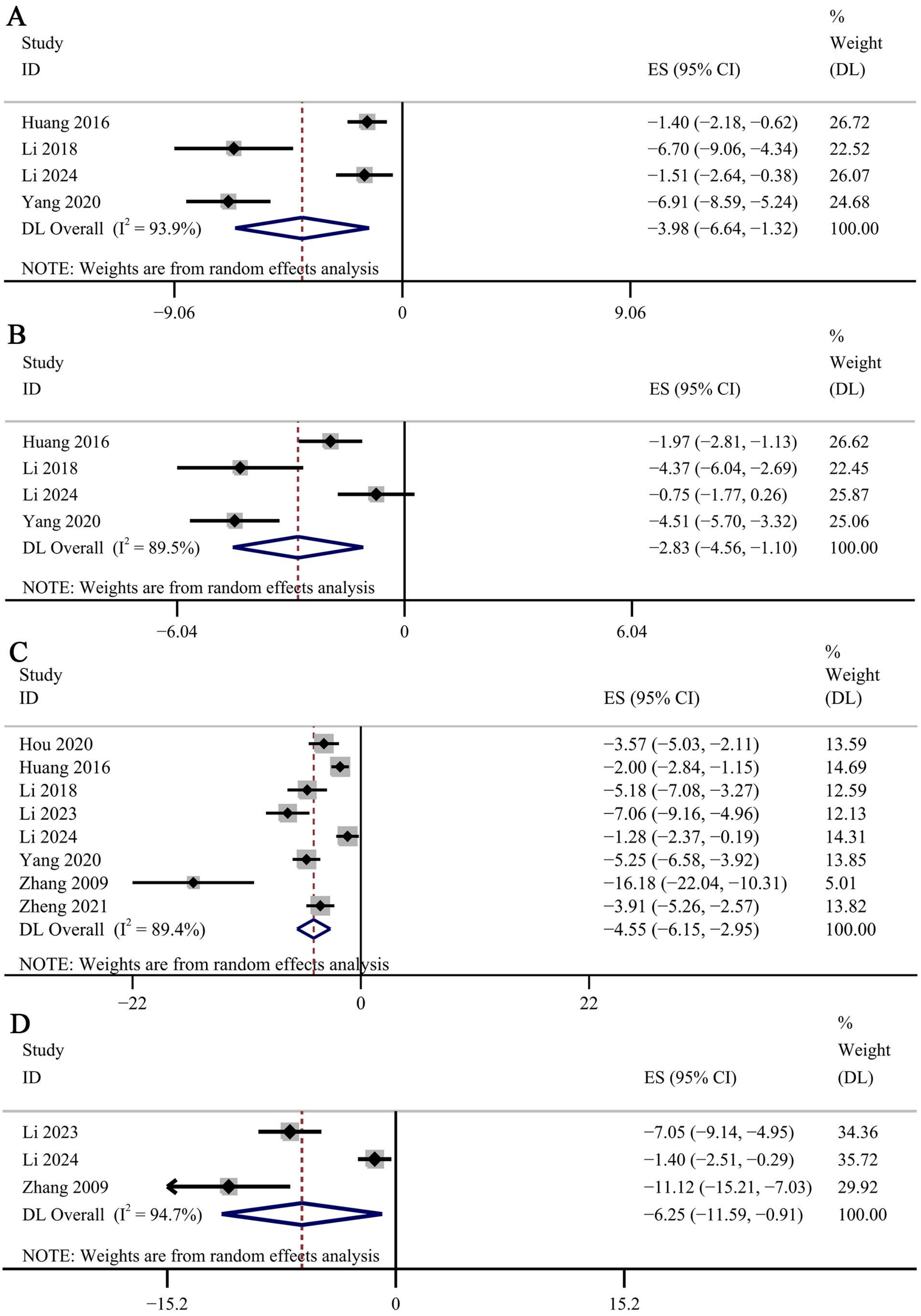

Supplementary Figure 1. Forest plots of IL-1β (A), IL-6 (B), TNF-α (C), and MCP-1 (D).

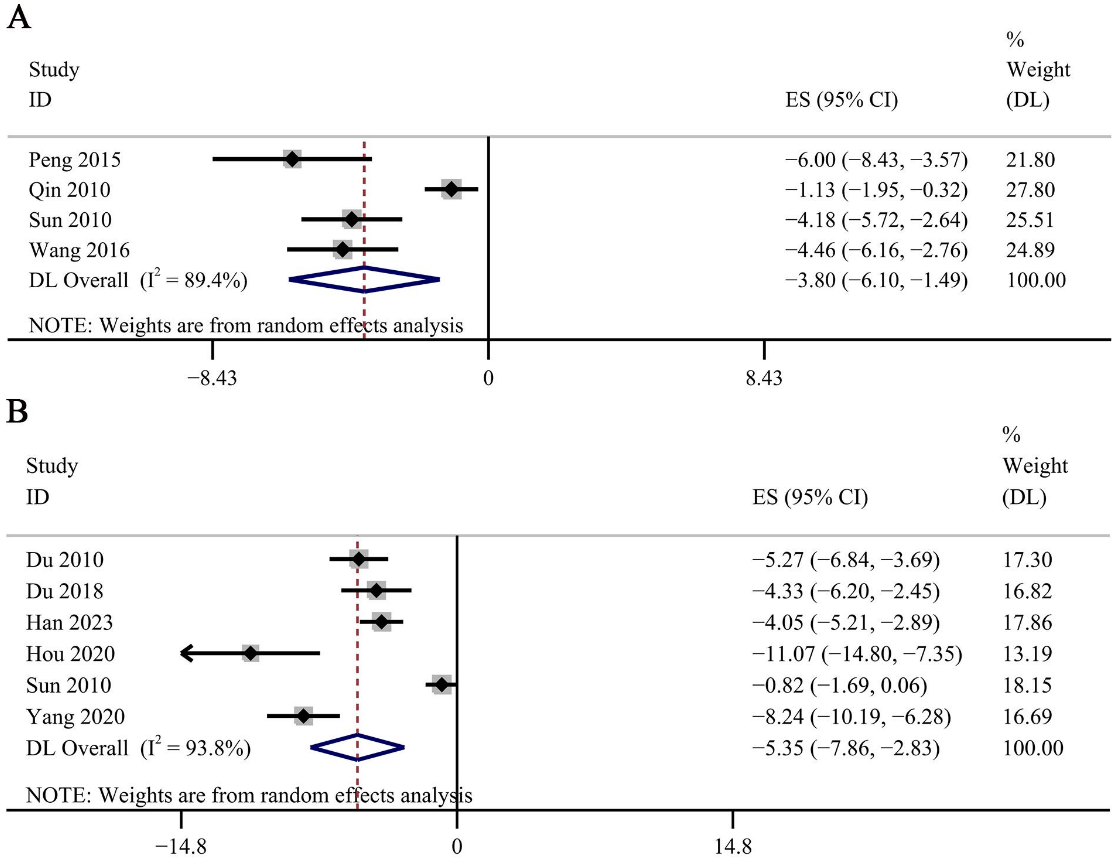

Supplementary Figure 2. Forest plots of TGF-β1 at protein level (A) and gene level (B).

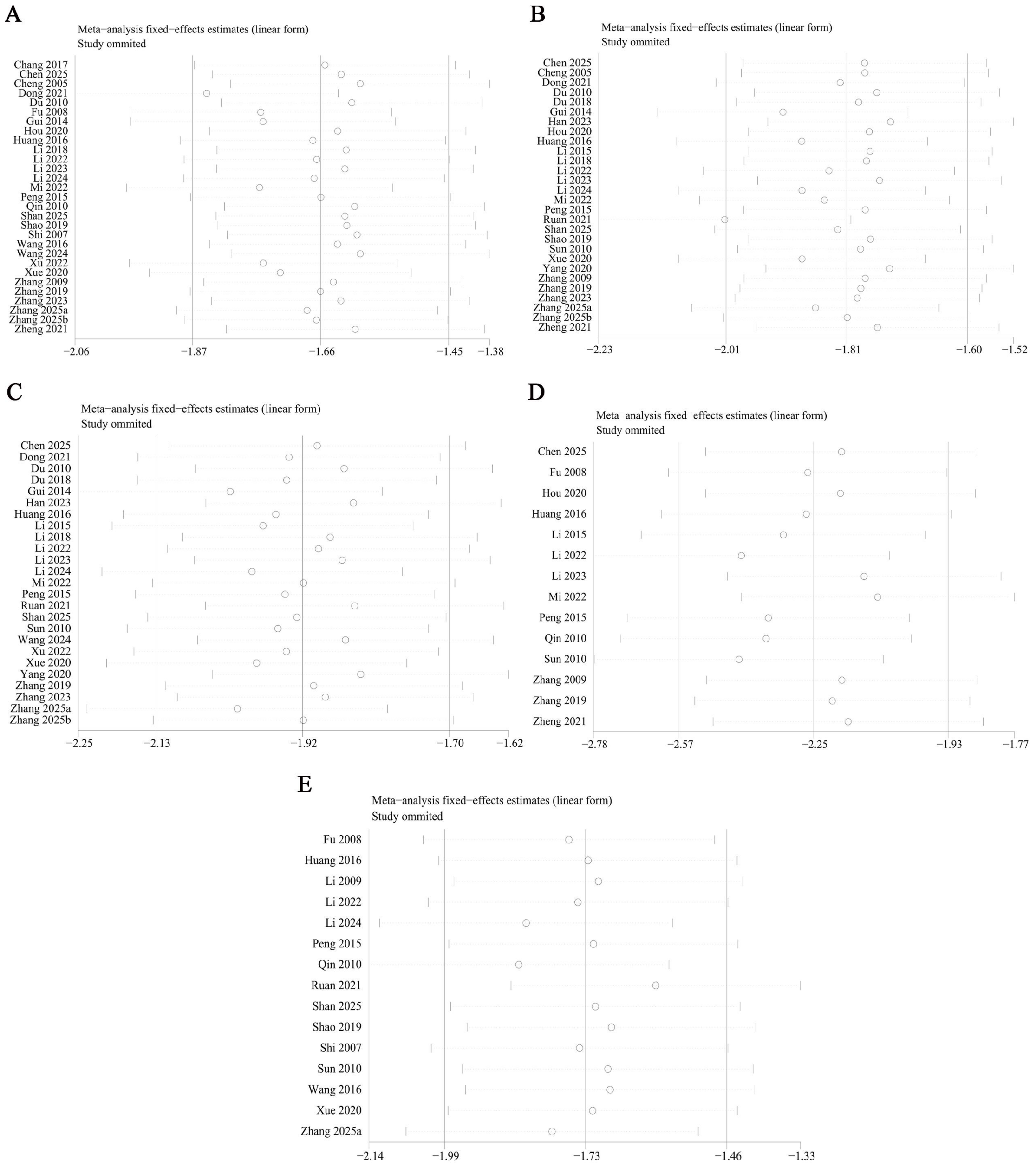

Supplementary Figure 3. Sensitivity Analysis of FBG (A), SCr (B), BUN (C), 24h UPro(D), and KI (E).
